# CAR‐Aptamers Enable Traceless Enrichment and Monitoring of CAR‐Positive Cells and Overcome Tumor Immune Escape

**DOI:** 10.1002/advs.202305566

**Published:** 2023-12-26

**Authors:** Hang Zhou, Tuersunayi Abudureheman, Wei‐Wei Zheng, Li‐Ting Yang, Jian‐Min Zhu, Ai‐Bin Liang, Cai‐Wen Duan, Kaiming Chen

**Affiliations:** ^1^ Key Laboratory of Pediatric Hematology and Oncology Ministry of Health, Pediatric Translational Medicine Institute, Shanghai Children's Medical Center Shanghai Jiao Tong University School of Medicine Shanghai 200127 China; ^2^ Fujian Branch of Shanghai Children's Medical Center, affiliated with Shanghai Jiaotong University School of Medicine and Fujian Children's Hospital Fuzhou Fujian 350005 China; ^3^ Department of Hematology, Tongji Hospital Tongji University School of Medicine Shanghai 200065 China; ^4^ Key Laboratory of Technical Evaluation of Fertility Regulation for Non‐human Primate, National Health Commission Fujian Maternity and Child Health Hospital Fuzhou Fujian 350122 China

**Keywords:** aptamers, CAR‐T cells, monitor, retargeting, traceless enrichment

## Abstract

Chimeric antigen receptor (CAR)‐positive cell therapy, specifically with anti‐CD19 CAR‐T (CAR19‐T) cells, achieves a high complete response during tumor treatment for hematological malignancies. Large‐scale production and application of CAR‐T therapy can be achieved by developing efficient and low‐cost enrichment methods for CAR‐T cells, expansion monitoring in vivo, and overcoming tumor escape. Here, novel CAR‐specific binding aptamers (CAR‐ap) to traceless sort CAR‐positive cells and obtain a high positive rate of CAR19‐T cells is identified. Additionally, CAR‐ap‐enriched CAR19‐T cells exhibit similar antitumor capacity as CAR‐ab (anti‐CAR antibody)‐enriched CAR‐T cells. Moreover, CAR‐ap accurately monitors the expansion of CAR19‐T cells in vivo and predicts the prognosis of CAR‐T treatment. Essentially, a novel class of stable CAR‐ap‐based bispecific circular aptamers (CAR‐bc‐ap) is constructed by linking CAR‐ap with a tumor surface antigen (TSA): protein tyrosine kinase 7 (PTK7) binding aptamer Sgc8. These CAR‐bc‐aps significantly enhance antitumor cytotoxicity with a loss of target antigens by retargeting CAR‐T cells to the tumor in vitro and in vivo. Overall, novel CAR‐aptamers are screened for traceless enrichment, monitoring of CAR‐positive cells, and overcoming tumor cell immune escape. This provides a low‐cost and high‐throughput approach for CAR‐positive cell‐based immunotherapy.

## Graphical abstract

a) The concept illustration of the CAR‐ap aptamer SELEX process. b) CAR‐ap aptamers used for traceless enrichment of CAR19‐T cells. c) CAR‐ap can specifically monitor CAR‐positive cells. d) CAR‐ap aptamers retarget CAR19‐T cells to CD19 antigen‐lost tumor cells.

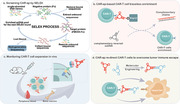

## 1 Introduction

Adoptive cell transfer (ACT) immunotherapies such as chimeric antigen receptor T (CAR‐T) or NK (CAR‐NK) cell therapy are the most promising treatment methods that have revolutionized cancer remedies.^[^ ^1^ ^]^ Several tumor surface antigens (TSAs) have been developed for CAR‐T therapeutic targets, including CD19, CD20, CD22, and B‐cell maturation antigens (BCMA).^[^ ^2^ ^]^ CD19 is the most widely used target in the field of CAR‐T cell therapy and was initially chosen as a CAR target over other B‐cell surface molecules (such as CD20 and CD22) owing to its relatively higher expression in most B‐cell malignancies.^[^ ^3^ ^]^ Anti‐CD19 CAR‐T (CAR19‐T) cell therapy achieves a 70%–90% complete response (CR) against relapsed and/or refractory CD19‐expressed B cell hematological malignancies.^[^ ^4^ ^]^ Currently, four CAR19‐T cell products were approved by the US Food and Drug Administration (FDA) for patients with relapsed or refractory B‐cell malignant diseases.^[^ ^5^ ^]^ Additionally, anti‐BCMA CAR‐T cell products were approved by the FDA to treat multiple myeloma.^[^ ^6^ ^]^

The treatment process of CAR‐T cells involves obtaining T cells from patients, expressing CAR with specific binding to tumor antigen in T cells, infusing expanded CAR‐T cells back into the patients, and monitoring CAR‐T treatment responses.^[^ ^7^ ^]^ CAR‐T cell manufacturing requires isolating autologous T cells from patients' peripheral blood mononuclear cells (PBMCs) and infecting them with lentivirus expressing CAR with specific binding to tumor antigens, such as CD19, CD20, and CD22. Only CAR‐positive T cells exert specific antitumor effects; CAR‐negative T cells are a byproduct. The packaging specifications and clinical dose of CAR‐T cell products are denoted in terms of the number of CAR‐positive T cells. Therefore, the positive rate of CAR transfection is a mandatory test for the quality control of CAR‐T cell products and is generally detected or enriched by flow cytometry after staining with the anti‐CAR antibody.^[^ ^8^ ^]^

CAR is a synthetic receptor that usually includes an antibody‐derived target binding extracellular domain, hinge region, transmembrane domain, and intracellular signal transduction domain. The expansion of CAR‐T cells in vivo is associated with their therapeutic efficacy and lethal toxic reactions. Monitoring transfused CAR‐T cells is crucial to ensure the therapeutic effect of CAR‐T cells and to prevent adverse effects in the clinical setting.^[^ ^9^ ^]^ Multiple assays were developed to detect the various regions of CAR19 by fluorescence‐assisted cell sorting (FACS), including CD19 protein, anti‐CAR19 antibodies, anti‐Fab antibodies, and Protein L. However, anti‐Fab antibodies and Protein L lack specificity for detection, while anti‐CAR antibodies are costly, and their production time is lengthy.^[^ ^10^ ^]^ In addition, these methods rely on large instruments and equipment and have low throughput. Therefore, developing low‐cost, traceless, and high throughput methods to enrich and monitor CAR‐positive cells is essential for the rapid advancement of CAR‐T cell therapy from preclinical models to clinical applications and to further expand the therapeutic outreach of CAR‐T cells.^[^ ^11^ ^]^

Aptamers are ssDNA or ssRNA with distinct secondary or tertiary structures that enable them to bind to corresponding molecular targets. They are typically screened by the SELEX (Systematic Evolution of Ligands by Exponential Enrichment) from a random library of 10^13^–10^16^ ssDNA or ssRNA molecules.^[^ ^12^ ^]^ Aptamers have limited immunogenicity and low molecular weight. Compared with antibodies, this results in fewer allergic reactions when applied to the body as a drug or molecular delivery vehicle.^[^ ^13^ ^]^ Aptamers, similar to antibodies, have high affinity and specificity binding to the target. Beyond that, aptamers have numerous unique advantages over antibodies, such as faster tumor tissue penetration and easier chemical modification.^[^ ^14^ ^]^ They have emerged as effective tools to recognize cancer‐related markers in targeted therapy, with great potential for diagnostic and therapeutic uses.^[^ ^15^ ^]^ They were recently used in CD8^+^ T cell traceless selection.^[^ ^16^ ^]^ However, the development and application of CAR‐specific binding aptamers are unreported.

Here, we identified several DNA aptamers (CAR‐aptamers or CAR‐ap) that specifically bind with the extracellular domain of CD19 CAR (FMC63, PDB: 7URV_D) using a protein‐SELEX procedure and demonstrate their excellent binding ability regardless of the preparation process of CAR‐T or CAR‐NK cells. We then used CAR‐ap to sort and enrich CAR‐positive cells in a traceless way in combination with its complementary reversal DNA sequence. In addition, these CAR‐aps can monitor the expansion of injected CAR‐T cells in the peripheral blood of mice, thereby predicting the treatment effectiveness of CAR‐T cells. Finally, we focused on the clinical problem of CD19 antigen deficiency after CAR‐T cell treatment and constructed CAR‐ap‐based bispecific circular aptamers that can re‐direct CAR‐T cells to tumor cells and execute the antitumor cytolysis function. Therefore, a novel type of CAR‐aptamer was identified that offers a highly efficient, accessible, and inexpensive alternative tool to enrich, monitor, and retarget CAR‐T cells for potential clinical‐scale cell therapy applications.

## 2 Results

### 2.1 Identification of CAR‐Binding Aptamers

**Figure 1:**
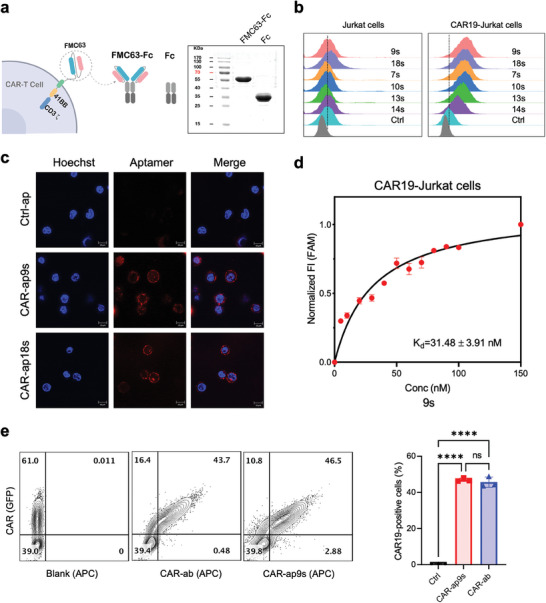
Screening of CAR‐aptamers by SELEX. a) FMC63‐Fc and Fc protein expression by HEK‐293T cells were confirmed by SDS‐PAGE stained with coomassie brilliant blue. b) The binding performance of aptamers, 7s, 9s, 10s, 13s, 14s, and 18s to Jurkat cells and CAR19‐Jurkat cells. c) Fluorescence confocal microscopy of CAR19‐Jurkat cells stained with Hoechst (blue) and cocultured with ctrl‐ap, CAR‐ap9s, and CAR‐ap18s (red). Scale bar, 20 µm. d) Flow cytometry binding curve of CAR‐ap9s to CAR19‐Jurkat cells. K_d_ values were calculated by averaging the individual regression values of the independent experiments. e) Flow cytometry assay of CAR‐ab and CAR‐ap9s binding to human PBMC‐derived GFP^+^ CAR19‐T cells. Left, flow cytometry plots representing three replicates. Right, a statistical graph of the CAR‐positive rate, defined as the percentage of GFP^+^ T cells that were also positive for antibody or aptamer binding.

**Scheme 1:**
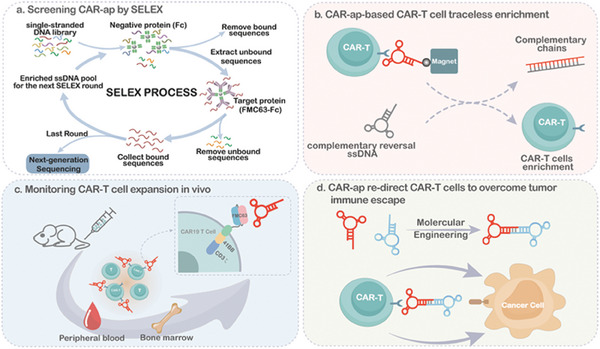
a) The concept illustration of the CAR‐ap aptamer SELEX process. b) CAR‐ap aptamers used for traceless enrichment of CAR19‐T cells. c) CAR‐ap could specifically monitor CAR‐positive cells. d) CAR‐ap aptamers are used for retargeting CAR19‐T cells to CD19 antigen‐lost tumor cells.

Anti‐CD19, CD20, CD22, CD7, and BCMA CAR‐T have been applied to treat B‐cell or T‐cell hematological tumors and multiple myeloma.^[^ ^2^, ^17^ ^]^ This study selected the classical anti‐CD19 CAR (CAR19) as a paradigm to screen CAR‐binding aptamers. Highly specific CAR19 aptamers were identified by adopting a protein‐SELEX procedure to specifically select ssDNA aptamers binding to the FMC63 protein (PDB: 7URV_D) using random sequences (10^15^ variants). The expressed sequences of FMC63‐Fc and Fc proteins were cloned into the pSB plasmid (Figure S1a, Supporting Information),^[^ ^18^ ^]^ expressed in HEK‐293T cells, and the proteins were purified (**Figure** **1**a). The ability of the FMC63 protein to bind to CD19‐positive target cells was validated (Figure S1b, Supporting Information). FMC63‐Fc and Fc protein were loaded onto protein A agarose resin during the screening process, wherein only the selected aptamers recognized the target FMC63 segment. Initially, the ssDNA sequence library was incubated with Fc protein (negative target) as a negative selection. The remaining DNA sequences in the supernatant were collected and incubated with FMC63‐Fc protein (positive target). The DNA sequences that bound to the positive target were collected and amplified for the next round of selection. The pool of aptamers underwent multiple rounds of negative/positive selection to reduce the DNA sequences combined with negative protein and increase the positive target‐specific sequences (**Scheme** **1**a). CAR19‐infected Jurkat (CAR19‐Jurkat) cells were prepared to verify the binding performance of the screening product, and their CAR19 expression was validated by flow cytometry using an anti‐CAR antibody (Figure S2, Supporting Information). Incubation with the 6th pool of aptamers (C6‐ap) produced a significant increase in fluorescence intensity in CAR19‐Jurkat cells (target cells) in comparison to the control aptamer (ctrl‐ap) but not in Jurkat cells (control cells) (Figure S3, Supporting Information). This indicated that a small part of ssDNA sequences was enriched with specific binding affinity to the CAR target.

The 6th pool of aptamers was cloned and sequenced, and six representative sequences were selected for further optimization and testing. The chosen aptamers were optimized for further experiments by designing shorter versions (Figure S4, Supporting Information). The 9s and 18s aptamers exhibited the best binding performance to CAR19‐Jurkat cells compared with all other aptamers (Figure 1b). The specificity of 9s and 18s to CAR19‐Jurkat cells was validated by confocal imaging (Figure 1c). The low apparent K_d_ value of 9s (K_d_ = 31.48 ± 3.91 nM) and 18s (K_d_ = 74.37 ± 8.10 nM) aptamers for affinity with CAR19‐Jurkat cells showed that they specifically recognize the target cells with high affinity according to flow cytometry measurements (Figure 1d; Figure S5, Supporting Information). These findings demonstrate that the 9s and 18s aptamers specifically bind to CAR19 or CAR19‐positive cells. We named these CAR‐binding aptamers as CAR‐aptamers, or CAR‐ap for short: CAR‐ap9s and CAR‐ap18s. The binding ability of CAR‐ap to other cell‐derived CAR19‐positive cells was determined by constructing CAR19‐GFP T cells from healthy donors (PBMCs‐derived CAR19‐T cells) and CAR19‐GFP NK cells from NK92 cells with CAR19‐GFP lentivirus (GFP indicates the expression of CAR19). We uncovered that CAR‐ap9s precisely binded with the PBMC‐derived CAR19‐positive (GFP^+^) T cells and CAR19‐positive (GFP^+^) NK92 cells (Figure 1e; Figure S6, Supporting Information). This was consistent with the positive rate detected with anti‐CAR19 antibodies (CAR‐ab). These results suggest that CAR‐aptamers may be used as powerful tools to detect CAR19‐positive cells.

### 2.2 CAR‐Aptamers Enable the Traceless Enrichment of CAR‐T Cells

**Figure 2:**
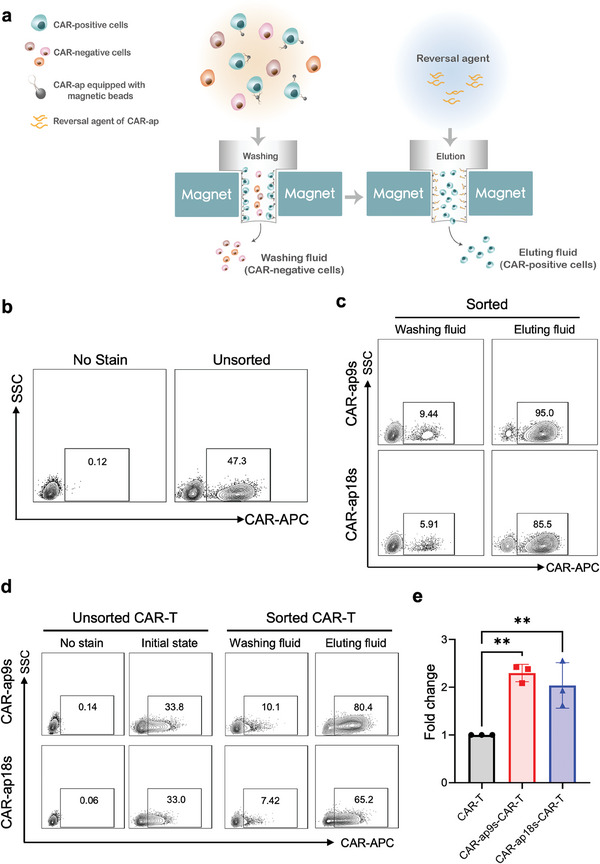
Traceless sorting of CAR‐positive cells using a CAR‐ap‐based strategy. a) Schematic illustration of the traceless sorting of CAR‐positive Jurkat or T cells using CAR‐ap aptamers. Biotin‐labeled CAR‐ap preloaded onto Streptavidin‐labeled magnetic beads incubated with different cellular fractions. Unlabeled CAR‐negative cells that were not captured were removed using the washing fluid. Captured CAR‐positive cells were released by magnetic beads after adding the reversal agent to the eluting fluid. b) Flow cytometry assay to detect the CAR‐positive rate of the cell sample mixed by CAR19‐Jurkat cells and Jurkat cells before CAR‐ap sorting. c) Flow cytometry assay to detect the CAR‐positive rate of the cell sample in the washing fluid and eluting fluid after CAR‐ap sorting. d) Flow cytometry assay to detect the CAR‐positive rate of human PBMC‐derived CAR19‐T cell samples before and after CAR‐ap sorting. The plots represent three independent experiments within human PBMCs from three healthy donors. e) Fold change in the proportion of CAR‐positive cells by flow cytometry analysis before and after sorting.

Reportedly, isolating aptamer‐bound cells and proteins requires aptamer reverse complementary sequences to change their secondary structure.^[^ ^16^, ^19^ ^]^ We used CAR‐ap9s and CAR‐ap18s to tracelessly sort the CAR19‐positive T cells (**Figure** **2**a). First, CAR19‐Jurkat cells were mixed with Jurkat cells to test the feasibility of CAR‐ap‐based isolation of CAR19‐positive cells precisely. CAR‐ap9s‐loaded magnetic beads almost entirely captured CAR19‐Jurkat cells and barely detected these cells in the washing fluid (Figure 2b and c). The CAR19‐Jurkat cells were obtained in the eluting fluid after adding a reversal agent (reverse complementary sequences of CAR‐ap9s, RCap9s). We further prepared three donors' PBMC‐derived CAR19‐T cells with between 20%–40% CAR‐positive T cells, which was consistent with the CAR‐positive rate of clinical application, for CAR‐ap‐based capture and enrichment of CAR19‐positive T cells. We observed that CAR‐ap‐based isolation significantly enriched CAR19‐positive T cells (Figure 2d) and elevated the ratio of CAR19‐T cells over two‐fold (Figure 2e). Moreover, microscopic examinations showed that the magnetic beads loaded with aptamers bind to target cells and form cell clusters that disappear after adding the reverse complementary sequence, and target cells were completely separated and recovered from the magnetic bead fraction (Figure S7, Supporting Information). These results demonstrate that CAR‐aptamers enable the traceless enrichment of CAR19‐T cells by using the aptamers' reverse complementary sequences (Scheme 1b).

### 2.3 CAR‐ap‐Enriched CAR‐T Cells Possess Antitumor Activity In Vitro and In Vivo

**Figure 3:**
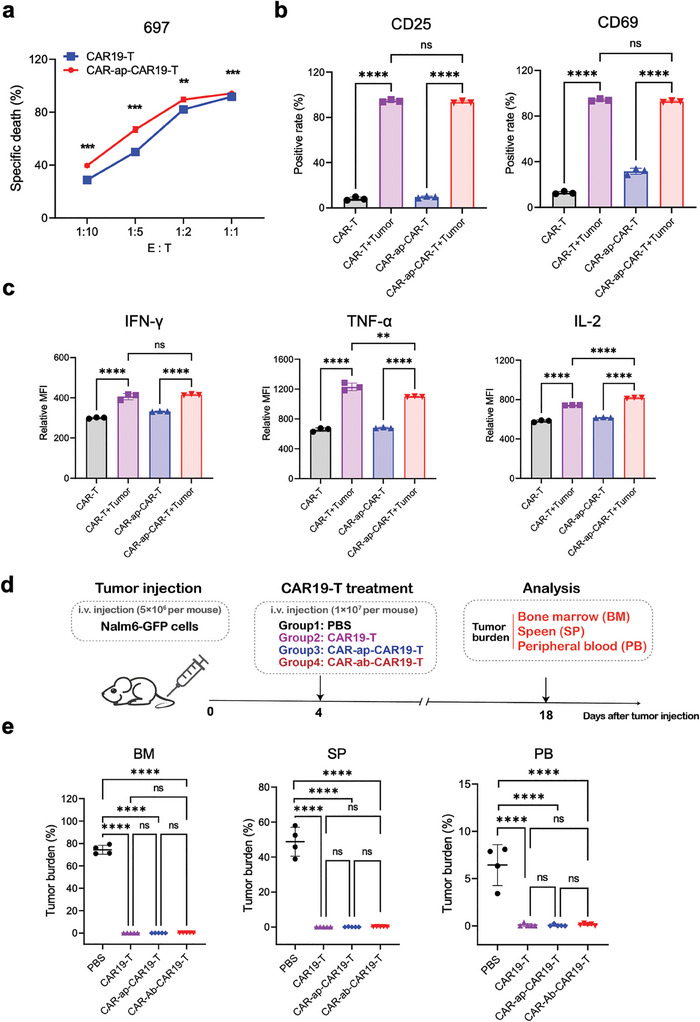
Functional validation of CAR19‐T cells sorted with CAR‐ap9s in vitro and in vivo. a) In vitro antitumor cytotoxicity of CAR19‐T cells and CAR‐ap9s‐CAR19‐T cells to 697 cells at different E:T ratios. The CAR‐positive rate was adjusted to the same level before the experiment. b) Flow cytometry assay to detect CD25 and CD69 expression of CAR19‐T cells and CAR‐ap9s‐CAR19‐T cells during in vitro antitumor cytotoxicity. c) Flow cytometry assay to detect IFN‐𝛾, TNF‐ɑ, and IL‐2 cytokine expression of CAR19‐T cells and CAR‐ap9s‐CAR19‐T cells during in vitro antitumor cytotoxicity. d) Schematic illustration of the Nalm6 mouse model treated with CAR19‐T cells, CAR‐ap9s‐CAR19‐T cells, and CAR‐ab‐CAR19‐T cells. The CAR‐positive rate was adjusted to the same level in each group before the experiment. e) Flow cytometry assay to measure the tumor burden (the rate of Nalm6‐GFP cells) in bone marrow (BM), spleen (SP), and peripheral blood (PB) of mice in each group. Statistical analysis was performed using ANOVA.

The antitumor cytotoxicity of CAR‐ap‐enriched CAR‐T cells was compared to that of the original CAR‐T cells to determine whether CAR‐ap‐based enrichment impacts the antitumor ability of CAR‐T cells. The positive rate of CAR‐ap‐enriched CAR‐T cells was readjusted to the ratio of original CAR‐T cells to compare the functions of the two types of cells quantitatively. Surprisingly, CAR‐ap‐enriched CAR‐T cells exhibited stronger killing ability than did the original cells as the ratio of effector cells to target cells (E:T) changed from 0.1 to 1 (**Figure** **3**a). We compared the activation level between the CAR‐ap‐enriched CAR‐T cells and the original CAR‐T cells by measuring CD25 and CD69 activation markers in CAR‐T cells after cocultured with Nalm6 cells, a cell line of B‐ALL. As anticipated, the incremental surface expression of CD25 and CD69 was similar in CAR‐ap‐enriched CAR‐T cells and original CAR‐T cells (Figure 3b).

Meanwhile, the effector cytokines tumor necrosis factor (TNF)‐α, interferon (IFN)‐γ, and interleukin (IL)‐2, secreted by both original CAR‐T cells and CAR‐ap‐enriched CAR‐T cells, could be significantly elevated when co‐incubated with tumor cells (Figure 3c). These findings suggest that the CAR‐ap‐based traceless enriched CAR‐T cells retain the same powerful antitumor ability as the original cells in vitro. This indicated that the CAR‐ap selection strategy did not have side effects on the antitumor capacity of CAR‐T cells in vitro.

GFP‐labeled Nalm6‐bearing mice were treated with CAR‐T cells that underwent different methodological enrichment processes, including antibody‐based flow sorting and CAR‐ap‐based magnetic bead sorting, to further investigate whether CAR‐ap‐enriched CAR‐T cells have a robust antitumor effect in vivo (Figure 3d). Mice were injected with 2 × 10^6^ Nalm6‐GFP cells and treated with 5 × 10^6^ untreated CAR‐T cells, CAR‐ab‐based flow cytometric‐enriched or CAR‐ap‐enriched CAR‐T cells 4 days later to test whether CAR‐ap‐based enrichment impairs the antitumor cytotoxicity of CAR‐T cells in vivo. The positive rate of CAR‐T cells from the different enrichment processes was adjusted to the same positive rate. Then, Nalm6‐GFP mice were treated with these CAR‐T cells. The tumor burden was measured in multiple mouse organs after treatment with CAR‐T cells for two weeks. Similar to treatment with the original CAR‐T cells, treatment with both CAR‐ap‐based sorted CAR‐T cells and CAR‐ab‐based sorted CAR‐T cells showed extraordinarily potent antitumor activity (Figure 3e). These results indicate that the CAR‐ap‐based cell sorting process does not impair the function of CAR‐T cells, which displayed excellent antitumor capabilities in vitro and in vivo.

### 2.4 CAR‐Aptamers Monitor the Expansion of CAR‐T Cells In Vivo

The number and proportion of CAR positives are the key characteristics of CAR‐T cell product activity. This is critical for the effective assessment of CAR‐T cell products since it is an indispensable part of the CAR‐T cell production process, the infusion back to the patient, and the monitoring of expansion in the patient. As a living cell drug, it is particularly important to monitor the expansion of CAR‐T cells in vivo since it provides valuable information about the efficacy and side effects of the treatment.^[^ ^11^, ^20^ ^]^ In addition, the lethal side effects associated with the expansion of CAR‐T cells (such as cytokine release syndrome) emphasize the importance of in vivo monitoring of CAR‐T cells.^[^ ^21^ ^]^ Initially, CFSE‐labeled CAR19‐T cells were injected into mice, and peripheral blood was subsequently withdrawn to measure the ratio of CAR19‐T cells and determine whether CAR‐aptamers can detect circulating CAR‐T cells in vivo (Figure S8a, Supporting Information). CAR‐ap9s (but not the control aptamer, ctrl‐ap) easily detect the presence of CAR19‐T cells in peripheral blood (PB). The ratio was consistent with the results detected with anti‐CAR19 antibodies (Figure S8b, Supporting Information). Furthermore, CAR‐ap9s‐stained CAR19‐T cells were observed by confocal microscopy, whereas the control aptamer‐stained CAR19‐T cells were not detected (Figure S8c, Supporting Information). These results indicate that CAR‐ap9s are well‐suited to monitor the circulation of CAR19‐T cells in peripheral blood.

**Figure 4:**
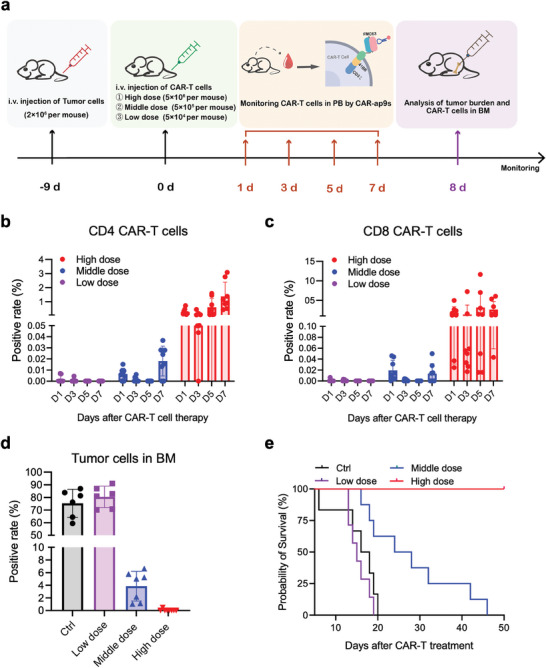
Monitoring of CAR19‐T cell expansion using CAR‐ap9s in a Nalm6 mouse model. a) Schematic illustration of the detection of CAR19‐T cell expansion in a CAR19‐T‐treated leukemia mouse model. b, c) Flow cytometry assay of the CD4^+^ CAR^+^ T cell positive rate (b) and CD8^+^ CAR^+^ T cell positive rate (c) using CAR‐ap9s in the peripheral blood of mice on day 1, day 3, day 5, and day 7 after treatment with CAR19‐T cells in three groups with different treatment doses (Low dose, Middle dose, and High dose). d) Flow cytometry assay of the tumor cell positive rate in the bone marrow (BM) of mice on day 8 after treatment with CAR19‐T cells in the control group and three groups with different treatment doses. e) Kaplan−Meier survival curves of the control group and three groups with different treatment doses.

We analyzed whether CAR‐ap9s could monitor the in vivo expansion of CAR‐T cells to offer clinical value in evaluating the treatment prognosis (**Figure** **4**a). Three doses of CAR‐T cells were used to treat Nalm6‐GFP leukemia mice and mimic the treatment response and recurrence of the disease. We then used CAR‐ap9s to continuously measure the ratio of CAR‐T cells in the peripheral blood of mice. Finally, bone marrow punctures were performed to detect tumor burden and observe the survival time of mice. Indeed, CAR‐ap9s truly gauged the expansion of CAR‐T cells in peripheral blood, the ratio of which is consistent with the dose gradient in different groups (Figure 4b,c; Figure S9a and b, Supporting Information).

Furthermore, the proportion of CD4 and CD8 CAR‐T cells were tracked in the peripheral blood of the high‐dose CAR‐T treated mice at different time points to distinctly visualize the expansion curve of CAR‐T cells in each mouse (Figure S9c and d, Supporting Information). A greater expansion of CAR‐T cells detected by CAR‐ap9s in high‐dose CAR‐T treated mice reduced the tumor burden and displayed the best therapeutic performance (Figure 4d; Figure S10, Supporting Information). Therefore, clinical efficacy was directly related to the expansion of CAR‐T cells. The survival curve also demonstrated that higher levels of CAR‐T cells detected by CAR‐ap9s in vivo correlated with longer mouse survival time (Figure 4e). These findings suggest that the specific binding of CAR‐ap to CARs could be an outstanding tool to monitor the in vivo expansion of CAR‐T cells during therapy, thereby predicting the therapeutic efficacy and disease prognosis (Scheme 1c).

### 2.5 Construction of a Bispecific Circular Aptamer With CAR‐ap and TSA‐Binding Aptamers

**Figure 5:**
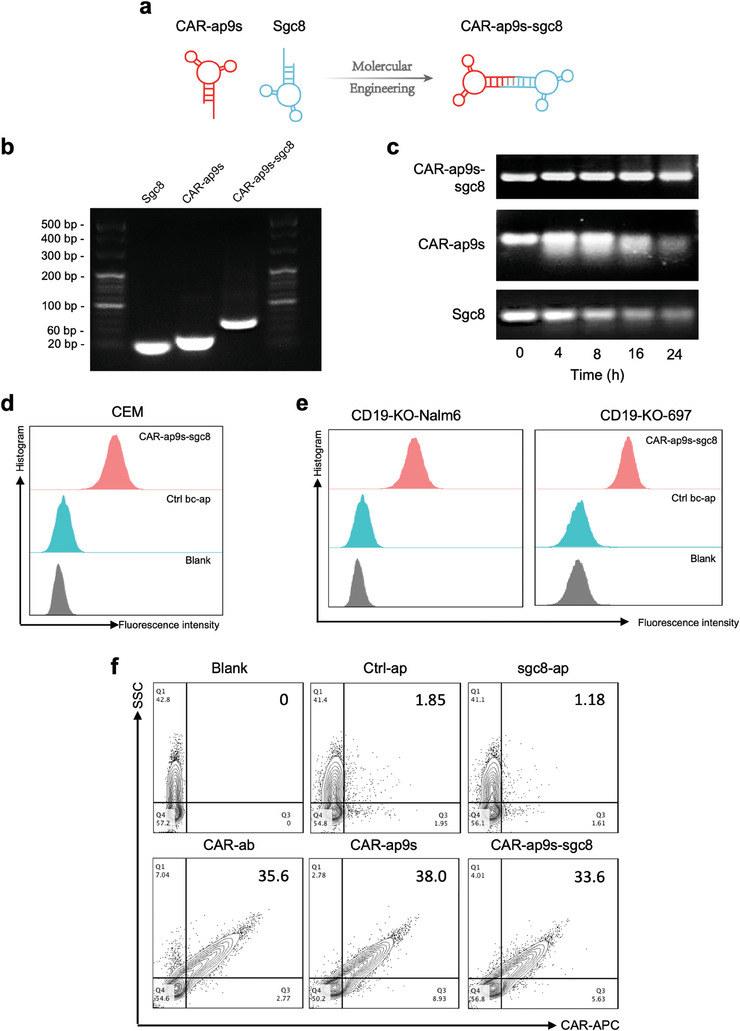
Construction and stability of the CAR‐ap‐based bispecific circular aptamer. a) Schematic illustrating the construction of bispecific circular aptamer CAR‐ap‐sgc8. b) Agarose gel electrophoresis of ssDNA CAR‐ap9s, ssDNA sgc8, and CAR‐ap9s‐sgc8. c) The stability of ssDNA CAR‐ap9s, ssDNA sgc8, and CAR‐ap9s‐sgc8 in FBS was determined by agarose gel electrophoresis at 37 °C for the different incubation times. d‐e) Flow cytometry assay to test the binding performance of CAR‐ap9s‐sgc8 to CEM cells and CD19‐KO BALL cells. f) Flow cytometry assay to measure the binding performance of CAR‐ab, ctrl‐ap, sgc8, CAR‐ap9s, and CAR‐ap9s‐sgc8 to CAR19‐T cells.

CAR‐T cell therapy treatment response is observed in most patients with hematological tumors; however, relapse has become an urgent problem. The loss of target antigen results in cancer cell resistance or evasion of CAR‐T cell therapy.^[^ ^22^ ^]^ New treatment strategies against other targets are reported to overcome CD19 negative relapse.^[^ ^22c^ ^]^ For instance, the design of dual/multi‐target CAR‐T cells is characterized by combined antigen targeting, wherein T cells simultaneously express two or more different CAR molecules to overcome tumor cell escape owing to the absence of a single target.^[^ ^23^ ^]^ The promising performance of aptamers in tumor‐targeted therapy led us to analyze whether CAR‐ap could retarget CAR19‐T cells to tumor cells with the loss of CD19 antigen, thus promoting antitumor efficacy.^[^ ^24^ ^]^ We first selected another tumor surface antigen (TSA) molecule (PTK7) widely expressed in various tumors and was investigated for oncology therapies.^[^ ^25^ ^]^ PTK7 is specifically recognized by aptamer Sgc8 to target tumor cells.^[^ ^26^ ^]^ PTK7 was expressed in multiple B‐ALL cell lines (Figure S11a and b, Supporting Information). In addition, confocal imaging illustrated that Sgc8 is bound with Nalm6 and 697 B‐ALL cells (Figure S11c, Supporting Information). Besides, we tested multiple healthy donors' PBMCs that did not bind to the Sgc8 aptamer to ensure its safety as a targeted agent (Figure S11d, Supporting Information). Then, we constructed a novel bispecific circular aptamer (bc‐ap) using T4 DNA ligase to conjugate CAR‐ap9s and Sgc8 to enable CAR‐T cells to re‐establish intercellular recognition with tumor cells (**Figure** **5**a). The two aptamers were assembled into one higher molecular weight bispecific circular aptamer, and this CAR‐ap‐based bispecific circular aptamer was called CAR‐bc‐ap, such as CAR‐ap9s‐sgc8 (Figure 5b).

Recent studies show that bispecific circular aptamers have improved stability since they lack free ends needed for nucleic acid degradation.^[^ ^27^ ^]^ Therefore, we tested the stability of ssDNA aptamers by incubating free CAR‐ap9s, Sgc8, and CAR‐ap9s‐sgc8 in 10% FBS at 37 °C for different time points. CAR‐ap9s and Sgc8 easily degraded in 10% FBS within 12 h, while CAR‐ap9s‐sgc8 was stable after incubation for 24 h (Figure 5c). CAR‐ap9s‐sgc8 was specifically bound to PTK7^+^ CCRF‐CEM cells (CD19 negative cells), a cell line of T‐cell leukemia (Figure 5d). Two CD19‐deficient cell lines were constructed to simulate the loss or downregulation of target antigens in B‐ALL, CD19‐KO‐Nalm6 (CD19 knock out Nalm6, CD19 negative cells), and CD19‐KO‐697 (CD19 knockout 697, CD19 negative cells) using the CRISPR/Cas9 system (Figure S12a, Supporting Information). Similarly, CAR‐ap9s‐sgc8 specifically binds to CD19‐KO‐Nalm6, CD19‐KO‐697 cells, and CAR‐T cells (Figure 5e and f). These data suggest that CAR‐bc‐ap efficiently targets tumor cells with lost or downregulated antigens.

### 2.6 CAR‐bc‐ap Overcomes Immune Escape by Retargeting CAR‐T Cells to Tumors in Vitro

**Figure 6:**
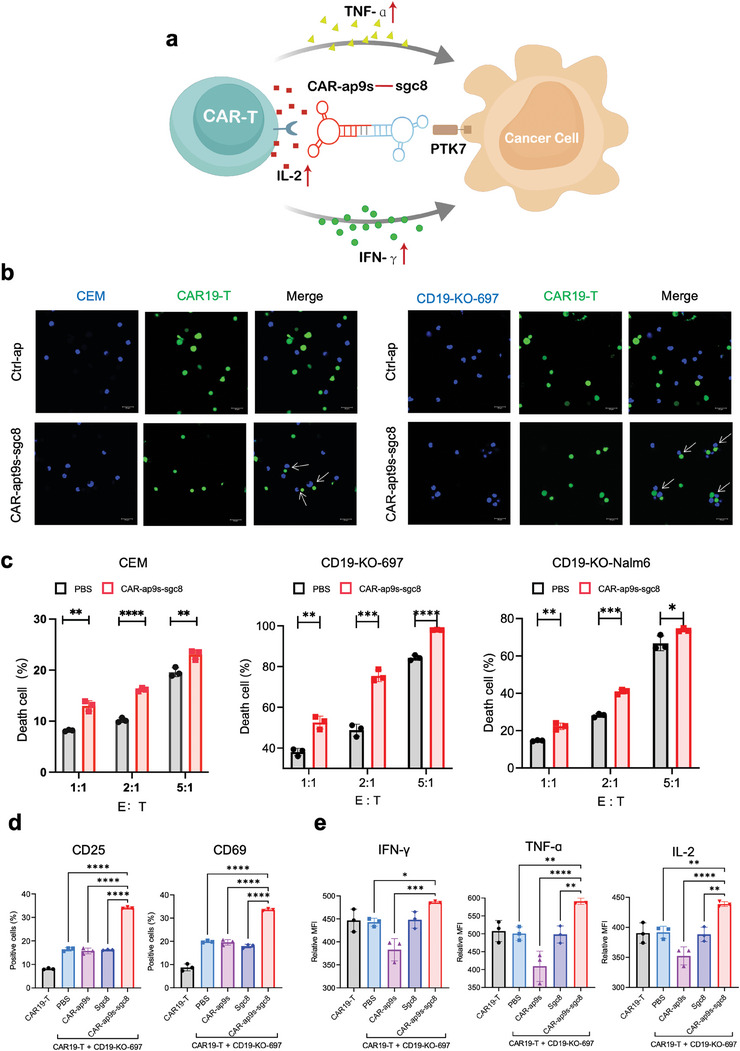
CAR‐ap9s‐sgc8 retargeted CAR19‐T cells to tumor cells with CD19 loss. a) Schematic illustration of CAR‐ap‐sgc8 mediated antitumor cytotoxicity of CAR19‐T cells to tumor cells with CD19 antigen loss. b) Fluorescence confocal microscopy of tumor cells stained with Hoechst (blue) co‐incubated with CFSE‐labeled CAR19‐T cells (green) in the presence of CAR‐apt9s(ap9s)‐sgc8 or ctrl‐ap. Left, CEM cells. Right, 697 cells. Scale bar, 20 µm. c) In vitro antitumor cytotoxicity of CAR19‐T cells to CEM, CD19‐KO‐697, and CD19‐KO‐Nalm6 cells at different E:T ratios in the presence or absence of CAR‐ap9s‐sgc8. d,e) Flow cytometry assay to detect active markers and cytokine expression of CAR19‐T cells during in vitro antitumor cytotoxicity in the presence of PBS, ssDNA CAR‐ap9s, ssDNA sgc8, and CAR‐ap9s‐sgc8. Statistical analysis was performed using ANOVA.

The above findings demonstrate that CAR‐bc‐ap maintains stability in a physiological setting and the binding ability to target cells. We further investigated whether CAR‐bc‐ap could facilitate the formation of intercellular connections between CAR‐T cells and tumor cells to re‐mediate tumor killing (**Figure** **6**a). CEM or CD19‐KO‐697 cells stained with Hoechst were co‐incubated with CFSE‐labeled CAR‐T cells with the CAR‐ap9s‐sgc8 or ctrl‐ap in binding buffer at 37 °C for 1 h to monitor intercellular junctions. Confocal microscopy imaging revealed that junctional cell‐cell complexes were observed after incubating with CAR‐ap9s‐sgc8, compared to cells cultured with ctrl‐ap (Figure 6b). This indicated that CAR‐ap9s‐sgc8 effectively mediated the cell‐cell interaction. CAR‐ap9s‐sgc8 or CAR‐ap18s‐sgc8 was cocultured with CAR19‐T cells and CD19‐KO‐Nalm6 or CD19‐KO‐697 cells, followed by apoptosis/necrosis assays using flow cytometry to investigate whether CAR‐bc‐ap overcomes the immune escape of tumor cells from CAR‐T killing owing to antigen loss. CAR‐ap9s‐sgc8 and CAR‐ap18s‐sgc8 promoted the antitumor capacity of CAR‐T cells when cocultured with CEM cells, while CAR‐bc‐ap alone had no significant effect on cell viability (Figure S12b, Supporting Information).

Furthermore, there was a significantly enhanced percentage of dead cells in the group treated with CAR‐ap9s‐sgc8 compared to those treated with PBS when CAR‐T cells were cocultured with CEM, CD19‐KO‐Nalm6, and CD19‐KO‐697 cells in different E:T ratios (Figure 6c). Meanwhile, there was no significant difference in the presence or absence of CAR‐ap9s‐sgc8 in transfected T cells killing CD19‐KO‐Nalm6 (Figure S12c, Supporting Information). Moreover, the levels of cell activation markers and cytokines of CAR‐T cells were much higher than those of any other treatment groups after co‐incubation with CD19‐KO‐Nalm6 cells in the presence of CAR‐ap9s‐sgc8 (Figure 6d and e). These findings suggest that CAR‐bc‐ap enhances the cytotoxicity of CAR‐T cells against tumor cells by increasing cell activation and cytokine secretion.

To further validate that the retargeting killing strategy of CAR‐bc‐ap is versatile, we utilized the MJ5C aptamer, which specifically binds to the PDL‐1 target,^[^ ^28^ ^]^ with CAR‐ap9s to construct a new bispecific circular aptamer, CAR‐ap9s‐MJ5C (Figure S13a, Supporting Information). The stability of circular CAR‐ap9s‐MJ5C was greatly improved in 10% FBS at 37°C compared to the single‐chain MJ5C aptamer (Figure S13b, Supporting Information). PDL‐1‐positive cell line SKBR3, which binds specifically to MJ5C aptamer (Figure S13c, Supporting Information), was used to detect CAR‐ap9s‐MJ5C‐mediated antitumor effects of CAR19‐T cells. The results showed that the killing efficiency of SKBR3 by CAR19‐T cells was significantly increased in the presence of CAR‐ap9s‐MJ5C (Figure S13d, Supporting Information). These results further demonstrate the great flexibility and versatility of the CAR‐bc‐ap strategy, providing new innovative ideas for tumor therapy.

### 2.7 CAR‐bc‐ap Overcomes Immune Escape by Retargeting CAR‐T Cells to Tumors in Vivo

**Figure 7:**
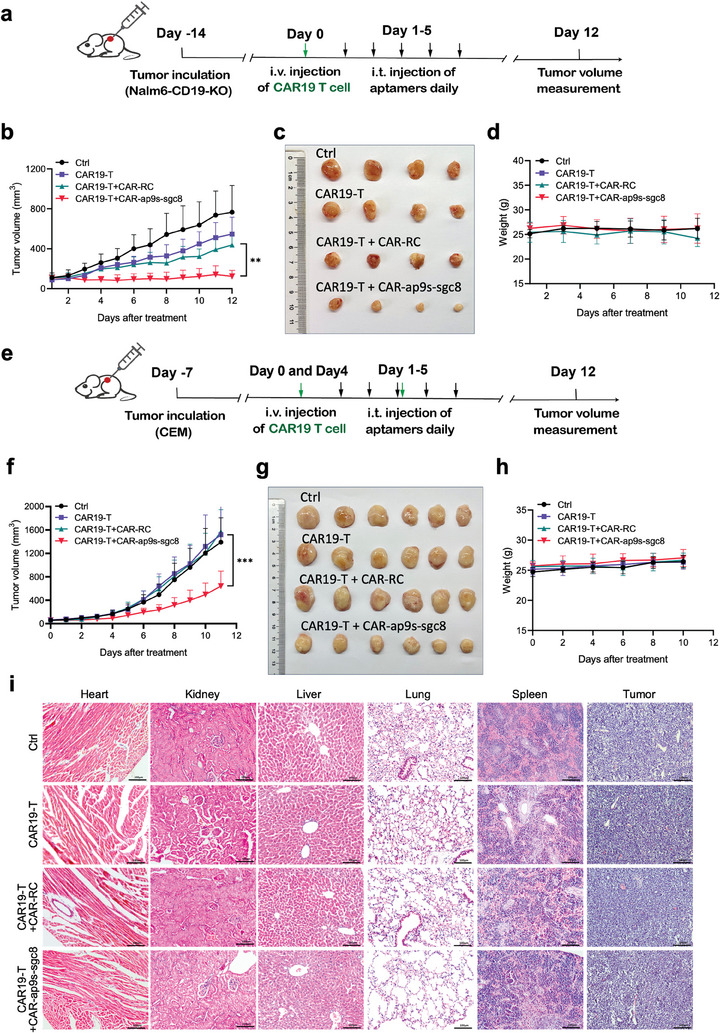
The potency of CAR‐ap9s‐sgc8‐mediated antitumor immunity of CAR19‐T cells in vivo. a) Schematic illustration of the therapeutic protocol used to investigate the function of CAR‐ap9s‐sgc8 mediating CAR‐T in CD19‐KO‐Nalm6 tumor xenografts. NSG mice (n = 4 per group) were inoculated in the flank with CD19‐KO‐Nalm6 tumor cells (1×10^7^) on day −14. After two weeks of tumor establishment, CAR19‐T cells (1×10^7^) were administered intravenously (i.v.) to tumor‐bearing NSG mice on day 0. Different groups of mice received different intratumoral (i.t.) treatments from day 1 to day 5 (50 µL of PBS for CTRL and CAR19‐T groups, bc‐ap9sRC‐sgc8RC (bc‐RC), or bc‐ap9s‐sgc8). b) Average tumor growth kinetics of mice receiving different treatments. Tumor volume (mm^3^) was monitored daily using the caliper method. Data are presented as means ±s.d. (n = 4). Statistical analyses were performed using two‐tailed paired Student's t‐tests (*p <0.05, **p<0.01, ***p <0.001, n.s., not significant). c) Ex vivo tumors in each group on day 12 after receiving various treatments as indicated. d) Body weight was monitored and recorded. Data are presented as means±s.d. (n = 4). e) Schematic illustration of the therapeutic protocol used to investigate the function of CAR‐ap9s‐sgc8 mediating CAR‐T in CCRF‐CEM tumor xenografts. NSG mice (n = 6 per group) were inoculated in the flank with CEM tumor cells (5×10^6^) on day −7. After allowing 1 week for tumor establishment, CAR19‐T cells (1×10^7^) were administered intravenously (i.v.) to tumor‐bearing NSG mice on days 0 and 3. Different groups of mice received different intratumoral (i.t.) treatments from day 1 to day 5 (50 µl of PBS for CTRL and CAR19‐T groups, bc‐RCap9s‐RCsgc8 (bc‐RC), or bc‐ap9s‐sgc8). f) Average tumor growth kinetics of mice under different treatments. Tumor volume (mm^3^) was monitored daily using the caliper method. Data are presented as means ±s.d. (n = 6). Statistical analyses were performed using a two‐tailed paired Student's t‐test (*p <0.05, **p<0.01, ***p <0.001, n.s., not significant). g) Ex vivo tumors in each group on day 11 after receiving indicated treatments. h) Body weight was monitored and recorded. Data are presented as means±s.d. (n = 6). i) H&E staining of tissue sections from CD19‐KO‐Nakm6 tumor xenografts after treatment. Scale bar: 100 µm.

Encouraged by the enhanced antitumor effects of the CAR‐bc‐ap strategy in vitro, we next investigated whether CAR‐bc‐ap could still mediate the antitumor effects of CAR‐T cells in vivo. CD19‐KO‐Nalm6 and CEM tumor‐bearing NSG mice were established by subcutaneously inoculating with CD19‐KO‐Nalm6 or CEM cells. After the tumor volume reached ≈100 mm^3^, CAR19‐T cells were infused intravenously (i.v.) into CD19‐KO‐Nalm6‐bearing NSG mice on day 0. Subsequently, PBS, CAR‐RCap9s‐RCsgc8 (CAR‐RC, as control agent), or CAR‐ap9s‐sgc8 aptamers were intratumorally injected daily for up to 5 days, and the tumor volumes were monitored daily until day 12 (**Figure** **7**a). The tumor growth was significantly inhibited in CD19‐KO‐Nalm6‐bearing mice treated with CAR19‐T cells plus CAR‐ap9s‐sgc8 compared to CAR19‐T cells plus CAR‐RC or CAR19‐T cells alone (Figure 7b and c). Similarly, only CAR19‐T cells plus CAR‐ap9s‐sgc8 suppressed the growth of tumors in the CEM mouse model (Figure 7e–g). Furthermore, the potential toxicity of CAR‐bc‐ap in vivo was also carefully analyzed. After receiving different treatments, the body weight of the mice was analyzed, and the major organs, including the tumor, liver, heart, spleen, lung, and kidney, were examined by histology. No noticeable body weight loss was observed (Figure 7d and h). Moreover, the H&E images showed no obvious histological differences between different groups, suggesting the excellent bio‐safety of our designed CAR‐bc‐ap (Figure 7i; Figure S14a, Supporting Information). To further demonstrate the specificity and re‐targetability of CAR‐ap9s‐sgc8‐mediated antitumor effects of CAR‐T cells, we constructed two circular aptamers, CAR‐RCap9s‐sgc8 and CAR‐ap9s‐RCsgc8 (designed as control), with only one side altered, and combined with CAR19‐T cells to treat tumor‐bearing NSG mice. The results showed that only the bispecific circular aptamer CAR‐ap9s‐sgc8 could mediate the antitumor effects of CAR19‐T cells (Figure S14b and c, Supporting Information). Taken together, our results illustrate that the antitumor efficacy of CAR‐T cells in vivo could be significantly improved by the CAR‐bc‐ap‐mediated retargeting activity of CAR‐T cells.

## 3 Discussion

CAR‐ap aptamers had a high affinity for the target and were easy to synthesize and modify, which made their properties conducive to subsequent applications. In addition, since the screening cycle of aptamers was shorter and easier than that of antibodies, screening the specific aptamers for CAR‐T cells for other targets, such as CD20, CD22, and BCMA, could be advanced without difficulties in the future. Accordingly, large‐scale manufacturing of aptamers and functional modification technologies was more mature, which was extremely favorable for clinical application in detection and therapy.^[^ ^29^ ^]^ While aptamers did draw attention for their numerous excellent properties, they had a flexible conformation and were therefore inherently dynamic. This often leads to attenuation of binding affinity, off‐target interactions, and susceptibility to exonucleases in biofluids, thus impeding the aptamer's use in biomedical applications.^[^ ^13^, ^30^ ^]^ There have been abundant reports on prolonging the half‐life of aptamers in vivo by increasing their stability in various ways,^[^ ^30^ ^]^ such as aptamer/albumin complexes,^[^ ^31^ ^]^ conformation‐stabilized aptamers with photochemically covalent lock,^[^ ^32^ ^]^ or screening for L‐DNA aptamers resistant to degradation,^[^ ^33^ ^]^ etc., which had broadened the development and application of aptamers in the field of detection and therapy.

The CAR‐ap aptamers performed well in both CAR19‐T cell enrichment and monitoring processes. They effectively increased the positivity rate of CAR19‐T cells and sensitively detected the expansion of CAR19‐T cells in peripheral blood. Chemically synthesized aptamers not only had comparable high sensitivity compared to antibodies used in conventional assays and cell sorting methods but also possess a number of advantages, such as faster batch synthesis, less batch‐to‐batch variability, easier chemical modification, better cost‐effectiveness, and longer shelf life.^[^ ^34^ ^]^ However, unmodified aptamers were susceptible to nuclease interaction and were not as efficiently detected as antibodies in complex environments such as serum. Besides, aptamers had multiple negative charges on their surfaces. They were prone to nonspecific binding to plasma proteins and high uptake by non‐target tissues, which could lead to undesired side effects and reduced efficacy. As a result, there also have been numerous reports of combining antibodies with aptamers to construct better assays or therapeutics.^[^ ^35^ ^]^

The aptamer‐based CAR‐T cell enrichment platform was the basis of a pattern that could be replicated and improved. While CAR‐T cells are collected, uninfected T cells can also be collected for secondary infection, increasing the total infection efficiency of fixed T cells. In addition, a balanced CD4:CD8 ratio of CAR‐T cells has been reported to both increase antitumor efficacy and, at the same time, reduce the dose of cells used by 5–100‐fold in CAR19‐T therapy.^[^ ^36^ ^]^ Thus, CAR‐ap aptamers used in combination with aptamers targeting CD8 and CD4 could easily realize a serial selection strategy for isolating CD4 CAR‐T cells and CD8 CAR‐T cells tracelessly from a single column and then precisely configure the right ratio for the treatment. In our enrichment platform, the CAR‐ap‐based magnetic bead sorting method is not yet optimal, and the CAR‐ap‐based microfluidic chip sorting mode can be used to capture CAR‐T cells more accurately in future studies. CAR19‐T cells that had been enriched with CAR‐ap aptamers performed even more strongly in killing assays in vitro, and this appeared to result from the slight activation of the CAR signaling pathway by CAR‐ap in combination with scFv of CAR (FMC63), which might be a potential risk for causing CAR19‐T cells to enter an early depletion state during long‐term treatment in vivo.

In the therapeutic process of retargeting CAR19‐T cells to tumor cells using bispecific circular aptamer‐mediated therapy, the aptamer played the role of bridging CAR‐T cells and tumor cells. In this strategy, the flexibility to change different tumor‐targeting aptamers was an advantage, thus eliminating tumors. In the future, multi‐targeted circular aptamers could even be utilized to enhance the tumor‐killing effect of CAR19‐T cells and further prevent off‐target effects. Currently, although the interest in therapeutic aptamers is significantly growing year after year, and several aptamer drugs are undergoing proof‐of‐concept studies and various stages of clinical trials, such as Pegaptanib (Macugen), Avacincaptad pegol (IZERVAY), and AS1411, most of them were still in preclinical research and far from applicability in clinical treatment. There was a significant gap between the antibodies already utilized in various clinical therapies. Although aptamers are smaller in mass and less immunogenic compared to antibodies and serious adverse effects are rarely observed in the clinic, caution is needed in their clinical application. The main sources of potential toxicity of aptamers include polyvalent anions on the surface of the aptamer, unintended tissue accumulation, risk due to chemical modifications, or nonspecific immune activation, especially if the aptamer drug was administered continuously.^[^ ^13b^ ^]^

For this study, we selected PTK7 as an alternative recognition site for tumor cells that had lost CD19 antigens. We used a bispecific cyclic aptamer to mediate CAR19‐T cells to re‐recognize and kill tumors. The use of bispecific cyclic aptamers to connect tumor cells to T cells or NK cells for antitumor effects has been reported.^[^ ^27^, ^37^ ^]^ However, there were no current clinical applications of bispecific circular aptamers. The undeniable potential risk was that the new targeting effect would likewise be attenuated or even off‐target again as the expression of PTK7 on tumor cells declines. The construction of multi‐target aptamers, similar to triple antibodies, has been reported and would effectively prevent potential off‐target effects due to single‐site deletions. Although the cyclic aptamer was substantially more stable in serum, it was therapeutically limited by its still small molecular weight, which was rapidly excreted via renal filtration, resulting in a very short circulation time. Further research is required to prolong the half‐life or reduce the side effects of bispecific circular aptamers due to their limitations for in vivo therapy. However, this innovative therapeutic concept offers a promising alternative approach to treating tumors.

In conclusion, we constructed an aptamer‐based platform for traceless sorting and monitoring CAR‐T cells by screening with high recognition efficiency to CAR‐positive cells, such as CAR‐T and CAR‐NK cells. We achieved traceless sorting and enrichment of CAR‐T/NK cells in an aptamer‐based manner. Then, we realized the retargeting of CAR‐T cells to tumor cells with missing antigens by constructing bispecific circular aptamers. This has significant implications for producing, detecting, and treating CAR‐positive cells. First, the CAR‐aptamers are characterized as operable, accessible, and affordable and have comparable capabilities to antibodies in terms of detecting CAR‐positive cells during preparation and peripheral blood after CAR‐T cell injection. The CAR‐ap‐mediated enrichment method can be used to obtain CAR‐T cell products with a high positive rate in a rapid and traceless way. This applies to the field since there is a high heterogeneity of infection efficiency of CAR‐T cells derived from patient‐derived PBMCs in clinical practice. Furthermore, CAR‐ap can be used to monitor the real‐time expansion of CAR‐T cells in vivo after infusing CAR‐T cells back into patients, thereby predicting the treatment effectiveness and side effects. Moreover, we selected suitable tumor‐targeting aptamers to build a highly stable bispecific circular aptamer with the CAR‐aptamers to retarget CAR‐T cells to tumor cells in the face of tumor cells that escape from CAR‐T cells due to antigen loss. Together, we discovered high‐affinity CAR‐specific aptamers for multiple aspects of CAR‐positive cell therapy technology. Our work drives the continuous updating of aptamer‐based assays and therapeutics that can lead to implementation into clinical workflows, thus increasing the accessibility of ACT immunotherapies, such as CAR‐T or CAR‐NK.

## 4 Experimental Section

### Materials and Chemicals

RPMI‐1640, DMEM, Opti‐MEM, and CTS AIM V SFM cell culture media were purchased from Gibco (USA). NK‐92 Cell Specialized Medium was purchased from Procell (China). Fetal bovine serum (FBS) was purchased from BioVision (USA). Penicillin‐streptomycin and 0.25% Trypsin‐EDTA were purchased from NCM Biotech (China). MgCl_2_ was purchased from Sigma (USA). The PBS, D‐PBS, Imidazole, Ni‐IDA‐Sefinose Resin 6FF (Settled Resin), and all DNA used in the selection were purchased from Sangon Biotech (China). Streptavidin Sepharose High Performance and rProtein A Sepharose FF were purchased from Cytiva (China). PCR Master Mix, Agarose, Goldview Nucleic Acid Gel Stain, and Liposomal Transfection Reagent were purchased from Yeasen Biotechnology (China). The anti‐human CD3 antibody and anti‐human CD28 antibody were purchased from SinoBiological (China). The Interleukin‐2 (IL‐2) was purchased from JSSH (China). APC‐conjugated anti‐human CD19 antibody, PE‐Cyanine7‐conjugated anti‐human CD4 antibody, APC‐eFluor 780‐conjugated anti‐human CD8a antibody, eFluor 450‐conjugated anti‐human CD25 antibody, PE‐conjugated anti‐human CD69 antibody, eFluor 450‐conjugated anti‐human IFN‐γ antibody, PE‐conjugated anti‐human TNF‐ɑ antibody, FITC conjugated anti‐human IL‐2 antibody, Hoechst 33 342, Fixation/Permeabilization Concentrate, Fixation/Perm Diluent, carboxyfluorescein diacetate succinimidyl ester (CFSE), Lipofectamine 3000 and 3 M Sodium Acetate were purchased from Thermo Fisher (USA). Alexa Fluor 647‐conjugated anti‐FMC63 scFv monoclonal antibody was purchased from Bioswan (China). PTK7 polyclonal antibody and Beta Actin polyclonal antibody were purchased from Proteintech (China). T4 DNA Ligase was purchased from NEB (USA). Streptavidin Magnetic Beads and DAPI Staining Solution were purchased from Beyotime (China). Human Lymphocyte Separation Medium was purchased from DAKEWE (China). BD GolgiStop protein transport inhibitor was purchased from BD Biosciences (USA). The hematoxylin‐eosin staining regent was from BASO (China).

### Cell Lines Culture and Human T Cells

Nalm6, CCRF‐CEM, 697, Jurkat, REH, RCH‐ACV, Ramos, NK92mi, and HEK‐293T cell lines were purchased from the American Type Culture Collection. The first four cells were cultured in PRMI‐1640 medium supplemented with 10% heat‐inactivated fetal bovine serum and 1% penicillin‐streptomycin. HEK‐293T cells were cultured in DMEM medium supplemented with 10% FBS and 1% penicillin‐streptomycin. NK92mi cells were cultured in NK‐92 Cell Specialized Medium with 200 Unit mL^−1^ IL‐2. All cells were maintained at 37 °C and 5% CO_2_. Peripheral blood mononuclear cells (PBMCs) were isolated from healthy volunteer blood using human PBMC isolation buffer and cultured in CTS AIM V SFM medium with 4% FBS and 300 Unit mL^−1^ IL‐2.

### Construction of CAR19‐Jurkat Cells and CAR19‐T Cells

The anti‐CD19 CAR vector was provided by Tongji Hospital. Lentiviral production was performed by plating 5 × 10^6^ HEK‐293T cells in 100 mm culture dishes with lentiviral vectors using Liposomal Transfection Reagent. The supernatant containing lentivirus was harvested at 48 and 72 h and concentrated by ultracentrifugation at 4 °C (50 000 × g for 2 h). The concentrated virus precipitate was resuspended in PBS and stored at −80 °C. PBMCs were stimulated for 48 h using anti‐human CD3 and anti‐human CD28 antibodies, and T cells were transduced with concentrated lentivirus using 10 µg mL^−1^ polybrene. Jurkat cells were transduced with lentivirus for 48 h to construct anti‐CD19 CAR‐expressing‐Jurkat cells. The ratio of CAR‐positive cells was detected using flow cytometry.

### Protein Production

FMC63‐Fc sequence was linked by FMC63 (PDB: 7URV_D) and Fc‐6H, and FMC63‐Fc and Fc‐6H were cloned into the protein expression vector pSB. 293T cells were transfected with a protein expression vector stored in our laboratory using Lipofectamine 3000, and stable expression was achieved by selection with 2 µg mL^−1^ puromycin. The proteins were purified from the culture supernatant using gravity chromatography columns filled with Ni‐IDA. Briefly, the culture media of 293T cells was centrifuged at 4 °C (3000 × g for 5 min), and the supernatant was loaded onto the columns. The bound protein column was washed with 20 and 40 mM imidazole and eluted with 200 and 500 mM imidazole (Imidazole dissolved using PBS). An Amicon Ultra‐4 10 K centrifugal filter device (MERCK, USA) was used to concentrate and dialyze the protein thrice in PBS.

The sequence of the Fc‐6H protein was as follows:

SSGDKTHTCPPCPAPELLGGPSVFLFPPKPKDTLMISRTPEVTCVVVDVSHEDPEVKFNWYVDGVEVHNAKTKPREEQYNSTYRVVSVLTVLHQDWLNGKEYKCKVSNKALPAPIEKTISKAKGQPREPQVYTLPPSRDELTKNQVSLTCLVKGFYPSDIAVEWESNGQPENNYKTTPPVLDSDGSFFLYSKLTVDKSRWQQGNVFSCSVMHEALHNHYTQKSLSLSPGKGGLPETGGLEHHHHHH

### In Vitro SELEX Procedures

The ssDNA library used in SELEX contained a central randomized sequence of 30 nucleotides (nt) flanked by 20‐nt sequences for primer hybridizing. The reverse primer was labeled with biotin to separate the sense strand from the antisense strand by streptavidin‐coated Sepharose beads for the next selection round. FMC63‐Fc‐6H protein was immobilized to rProtein A agarose resin by Fc binding with Protein A reacted for 2 h at 25 °C with gentle shaking. The FMC63‐Fc‐6H‐coated beads were then washed three times and resuspended in PBS. The first round included an initial DNA library consisting of 5 nmol randomized oligonucleotides as a starting pool. Later rounds used 200 to 50 pmol sample that was amplified from the previous pool. In each round, the DNA pool in binding buffer (D‐PBS with 5 mM Mg^2+^) was heated at 95 °C for 5 min, followed by rapid cooling on ice for 5 min. Beads were washed with 0.5 mL D‐PBS three times before use. The empty rProtein A agarose resin beads or Fc‐6H coated beads were incubated with the DNA pool for gradually increased time (from 20 to 40 min) each round, and the FMC63‐Fc‐6H‐coated beads were incubated with the DNA pool for gradually decreasing time (from 50 to 10 min) each round at 37 °C. The FMC63‐Fc‐6H‐coated beads were washed with washing buffer (D‐PBS) and were used for PCR amplification. The number of optimized PCR amplification cycles for each round was confirmed by agarose gel electrophoresis. Streptavidin Sepharose High‐Performance beads were used to isolate the PCR products from the PCR reaction mixture. The biotinylated antisense DNA was then separated and eluted with 200 mM NaOH. Finally, the ssDNA was purified using 10% 3 M NaAc and 2.5 times ethanol.

### Sequence Truncation of the Selected Aptamers

dsDNA was generated by PCR from the ssDNA pool and ligated with the pMD‐19 T vector (Takara, China). The clones from the pool were sent for sequencing. The selected aptamers were truncated based on their secondary structures, as the Integrated DNA Technologies OligoAnalyzer Tool predicted. Shorter versions of each selected aptamer were synthesized and purified as described above. As previously described, their binding abilities were evaluated by FACS.

### Polymerase Chain Reaction (PCR)

The ssDNA pool of each round of selection was enriched, and dsDNA was generated by PCR in 50 µL reactions consisting of 25 µL 2 × PCR MasterMix, 5.0 µL eluted ssDNA template, 2.5 µL forward primer, 2.5 µL reverse primer and 15 µL deionized water. Amplification consisted of a pre‐denaturation cycle at 94 °C for 5 min, followed by denaturation at 94 °C for 30 s, annealing at 56 °C for 30 s, and extension at 72 °C for 20 s. One sample was taken out for amplification analysis at each of the 6, 8, 10, and 12 cycles. All remaining samples underwent a final extension cycle at 72 °C for 2 min. dsDNA was generated via symmetry PCR. Then, PCR was employed to produce sufficient ssDNA for affinity assays.

### Flow Cytometry Analysis with Antibodies

Anti‐CD19 CAR expression was assessed using the procedure described below. CAR‐Jurkat cells and CAR‐T cells were washed with PBS and resuspended in PBS. Then, cells were stained with Alexa Fluor 647‐conjugated rabbit anti‐FMC63 scFv monoclonal antibody at 4 °C for 30 min, then detected after washing with PBS. CD25 and CD69 expression was tested by staining cells with PE/Cy7‐conjugated anti‐human CD4 antibody, APC ef780‐conjugated anti‐human CD8 antibody, BV421‐conjugated anti‐human CD25, and PE‐conjugated anti‐human CD69 antibody, with Alexa Fluor 647‐conjugated rabbit anti‐FMC63 scFv monoclonal antibody at 4 °C for 30 min. Intracellular cytokine staining involved culturing cells for 18 h, adding BD GolgiStop protein transport inhibitor, and continuously culturing for another 6 h. The cells were stained with anti‐human CD4, anti‐human CD8, and anti‐FMC63 scFv antibodies for 30 min at 4 °C. Next, cells were fixed and permeabilized with the foxp3 transcription factor staining buffer set. Cells then were stained with eFluor 450‐conjugated anti‐human IFN‐γ antibody, PE‐conjugated anti‐human TNFα, and FITC‐conjugated anti‐human IL‐2 antibody at 4 °C for 30 min. Stained samples were analyzed with a flow cytometer (BD FACS Canto II, USA), and data were analyzed using FlowJo V software.

### Aptamer Binding to Living Cells

The binding ability of CAR‐ap aptamers with Jurkat‐CAR cells or CAR19‐T cells was evaluated. Cells (≈10^5^) were suspended in 200 µL binding buffer. Then, different CAR‐ap aptamers were added and incubated at 37 °C for 30 min. Cells were washed twice and observed using a confocal microscope (Leica TCS SP8, Germany) or analyzed by flow cytometry, and the data was analyzed using FlowJo V software.

### Traceless Selection of CAR‐expressing Cells

CAR‐ap9s‐biotin and CAR‐ap18s‐biotin aptamer were cocultured overnight with Streptavidin magnetic beads at 4 °C under gentle rotation. The aptamer‐conjugated beads were washed twice to remove unconjugated aptamer, then added with 1 × 10^7^ original mixed cells and incubated for another 30 min at 37 °C under gentle rotation. Cells conjugated with aptamer‐bound beads were washed using the binding buffer, and the washing fluid was collected for detection. Afterward, a 5‐fold reverse complementary chain agent was applied to the cells labeled with aptamer‐functionalized microbeads captured by the magnet at 25 °C under gentle rotation for 40 min. Then, the mixed fractions from the previous step were separated from the magnetic beads and cells using an eluting fluid. Similarly, the eluting fluid containing the cell fractions was collected for assay.

### Cell−Cell Interaction Efficiency

Jurkat‐CAR cells and CCRF‐CEM cells or 697 cells were washed with PBS buffer, incubated with carboxyfluorescein diacetate succinimidyl ester (CFSE) and Hoechst 33 342 for 15 min at 37 °C, washed three times with PBS to remove the unstained solution. Two types of cells were mixed with ctrl aptamer or circular bispecific (cb)‐aptamer at 4 °C under gentle rotation for 2 h. The images of junctional cell‐cell complexes were observed by confocal microscopy.

### Construction of the Bispecific Circular Aptamer

Two different sequences were dissolved in 1 × T4 ligase buffer at 95 °C for 5 min, quickly chilled to 16 °C, and the hybridized DNA was incubated with T4 DNA ligase at 16 °C overnight to form the bispecific circular aptamers. A 2.5‐fold volume of anhydrous ethanol and one‐tenth volume of sodium acetate was added to the DNA solution and incubated at −80 °C for 2 h. The sample was centrifuged at 21 000 x g for 10 min at 4 °C, and the precipitate of bc‐aptamers was washed with 75% ethanol. Finally, the DNA concentration was quantified using Nanodrop 3000.

### Stability Analysis of Bispecific Circular Aptamers

CAR‐ap9s aptamer, sgc8 aptamer, and CAR‐ap9s‐sgc8 cb‐aptamer were incubated with RPMI‐1640 media containing 10% FBS at various times (0, 4, 8, 16, and 24 h). Samples were heated at 95 °C for 5 min to denature active enzymes, then chilled on ice and analyzed using UV−vis spectrometry to determine sequence integrity and stability.

### In Vitro Study of Cellular Cytotoxicity

CCRF‐CEM, CD19‐KO‐Nalm6, and CD19‐KO‐697 cells were first stained with 0.25 µM CFSE. Then, these cells were cocultured with anti‐CD19 CAR‐T cells in 96‐well plates treated with different concentrations of cb‐aptamer at 37° under 5% CO_2_ for 24 h. Finally, the cells were stained with 5 µg mL^−1^ 4,6‐diamidino‐2‐phenylindole (DAPI) and analyzed by fluorescence confocal microscopy within 5 min. CFSE^+^/DAPI^+^ cells were counted as lysed cells.

### Western Blotting Analysis

CCRF‐CEM, Nalm6, 697, REH, RCH‐ACV, and Ramos cells (≈10^6^) were lysed in 30 µL SDS Lysis Buffer, and the mixture was denatured by heating at 100 °C for 10 min and chilling at 25 °C. Next, samples were electrophoresed by SDS‐PAGE gel, transferred to the NC membrane, and blocked by 5% silk milk for 1 h at 25 °C. Last, after incubating with PTK7 antibody and Beta Actin antibody at 4 °C overnight and washing three times with PBST (PBS with 1% tween‐20), the NC membrane was imaged using the ChemiDoc Imaging System (BIO‐RAD, USA).

### Detection of CAR19‐T Cell Expansion Using CAR‐ap in a Nalm6 Mouse Model

Severely immunocompromised NOD.Cg‐*Prkdc^scid^Il2rg^em1Smoc^* (M‐NSG) mice were purchased from the Shanghai Model Organisms Center, and all animal work described in this article complied with animal ethics and welfare standards. In the pre‐experiment, 10^7^ CFSE‐labeled CAR19‐T cells were injected into mice, and peripheral blood was collected for detection by CAR‐ap 2 h later. In the model of leukemia treated with CAR19‐T cells, mice were inoculated with 2 × 10^6^ Nalm6‐DesRed cells in 200 µL PBS by tail vein injection and randomly divided into four groups on day 9 to receive treatment. The control group (n = 7) was injected with PBS, and the other three groups were injected with high (5 × 10^6^, n = 8), medium (5 × 10^5^, n = 8), and low doses (5 × 10^4^, n = 7) of CAR19‐GFP T cells. Peripheral blood was collected from mice on days 1, 3, 5, and 7, and bone marrow was collected on day 8 after treatment. Peripheral and bone marrow blood are first processed for erythrocyte lysis using ACK lysis buffer, then stained with antibodies and CAR‐ap aptamers. The cells were observed by confocal microscopy or analyzed by flow cytometry using FlowJo V software.

### In Vivo CAR‐T Cell Treatment of the Nalm6 Mouse Model

The influence of the aptamer‐mediated traceless capture method on CAR19‐T cell antitumor capability was assessed by inoculating mice with 2 × 10^6^ Nalm6‐GFP cells in 200 µL PBS by tail vein injection. The mice were randomly divided into four groups after one week: Group 1: Intravenous injection of PBS buffer, n = 4; Group 2: Intravenous injection of 1 × 10^7^ original CAR19‐T cells; Group 3: Intravenous injection of 1 × 10^7^ CAR19‐T cells through traceless aptamer‐based isolation with the positive rate adjusted to match the original CAR19‐T cells; Group 4: Intravenous injection of 1 × 10^7^ CAR19‐T cells through anti‐FMC63 antibody‐based isolation with the positive rate adjusted to match the original CART cells. Two weeks later, the mice were euthanized, and the tumor burden was detected in the femur, liver, and spleen by flow cytometry.

### In Vivo bc‐Aptamer‐mediated CAR‐T Cell Treatment of the CD19‐KO‐Nalm6 Mouse Model and CEM Mouse Model

To establish a human tumor xenograft model, NSG mice were subcutaneously injected with cancer cells (1 × 10^7^ CD19‐KO‐Nalm6 cells or 5 × 10^6^ CEM cells) in the flank. When tumors became visible, their sizes were measured with a digital caliper every day, and the tumor areas were calculated in square millimeters (length × width× width/2). For the CD19‐KO‐Nalm6 mouse model, after 2 weeks of tumor implantation, each mouse received 1 × 10^7^ CAR19‐T cells and was injected through the tail vein (i.v.). Following this, depending on the experimental set‐up, 50 µL reagents (10 µM bc‐RCap9s‐RCsgc8, 10 µM bc‐RCap9s‐sgc8, 10 µM bc‐ap9s‐RCsgc8, and 10 µM bc‐ap9s‐sgc8) or 50 µL PBS (control) were intratumorally (i.t.) injected into each tumor daily for the next five days. For the CEM mouse model, on the first and third days, when the tumor is visible as a well‐formed tumor, each mouse received 1 × 10^7^ CAR19‐T cells and was injected through the tail vein (i.v.). Depending on the experimental set‐up, 50 µL reagents (10 µM bc‐RCap9s‐RCsgc8 and 10 µM bc‐ap9s‐sgc8) or 50 µl PBS (control) were intratumorally (i.t.) injected into each tumor daily on day 1 to day 5. Mice were euthanized for tumor isolation on day 12.

### Toxicity Assessment

To evaluate the potential side effects, the body weights of mice that received different treatments were recorded every 2 days. The mice were sacrificed after receiving different treatments to examine histological changes and assess the long‐term systematic toxicity. The major organs and the tumors were isolated and sliced to be stained with hematoxylin and eosin (H&E), followed by analysis using a positive binocular biomicroscope (DM6 B, Leica).

### Statistical Analysis

Statistical analysis of the data was performed using one‐way analysis of variance (ANOVA) to assess the differences between the different groups. All curve fitting was performed using Prism 9 (Graphpad), and P values are reported (not significant = p> 0.05, *p < 0.05, **p < 0.01, ***p < 0.001, ****p < 0.0001) and marked on the figures. All data are shown as means ± SD from at least three experiments.

## Conflict of Interest

The authors declare no conflict of interest.

## Author Contributions

H.Z. and T.A. contributed equally to this work. H.Z. and T.A. designed and performed the experiments, interpreted the results, and prepared the manuscript. W.‐W. Z., L.‐T. Y., and J.‐M. Z. performed some of the experiments. A.‐B.L. provided advice and reviewed the manuscript. K.C. and C.‐W.D. conceived of the study, designed experiments, supervised research, and wrote the manuscript.

## Supporting information

Supporting Information

## Data Availability

The data that support the findings of this study are available from the corresponding author upon reasonable request.
