# Supplementary material for: CAR‐Aptamers Enable Traceless Enrichment and Monitoring of CAR‐Positive Cells and Overcome Tumor Immune Escape
Source: Adv Sci (Weinh). 2023 Dec 26;11(10):2305566. doi: 10.1002/advs.202305566 (PMC10933668; doi:10.1002/advs.202305566)
Supplement: Supplementary file 1 — Supporting Information [file ADVS-11-2305566-s001.pdf]

ADVANCED SCIENCE
Open Access

Supporting Information

for *Adv. Sci.*, DOI 10.1002/advs.202305566

CAR-Aptamers Enable Traceless Enrichment and Monitoring of CAR-Positive Cells and Overcome Tumor Immune Escape

Hang Zhou, Tuersunayi Abudureheman, Wei-Wei Zheng, Li-Ting Yang, Jian-Min Zhu, Ai-Bin Liang, Cai-Wen Duan and Kaiming Chen**

Supporting Information
©Wiley-VCH 2021
69451 Weinheim, Germany

CAR-aptamers Enable Traceless Enrichment and Monitoring of CAR-positive Cells and Overcome Tumor Immune Escape

Hang Zhou, Tuersunayi Abudureheman, Wei-Wei Zheng, Li-Ting Yang, Jian-Min Zhu, Ai-Bin Liang, Cai-Wen Duan*, Kaiming Chen*

Contents

Supporting Results 1

Table S1. Sequences in this work... 14

Table S3. Sequences of CRISPR/cas9 sgRNA for CD19 knockout 17

Supporting Results

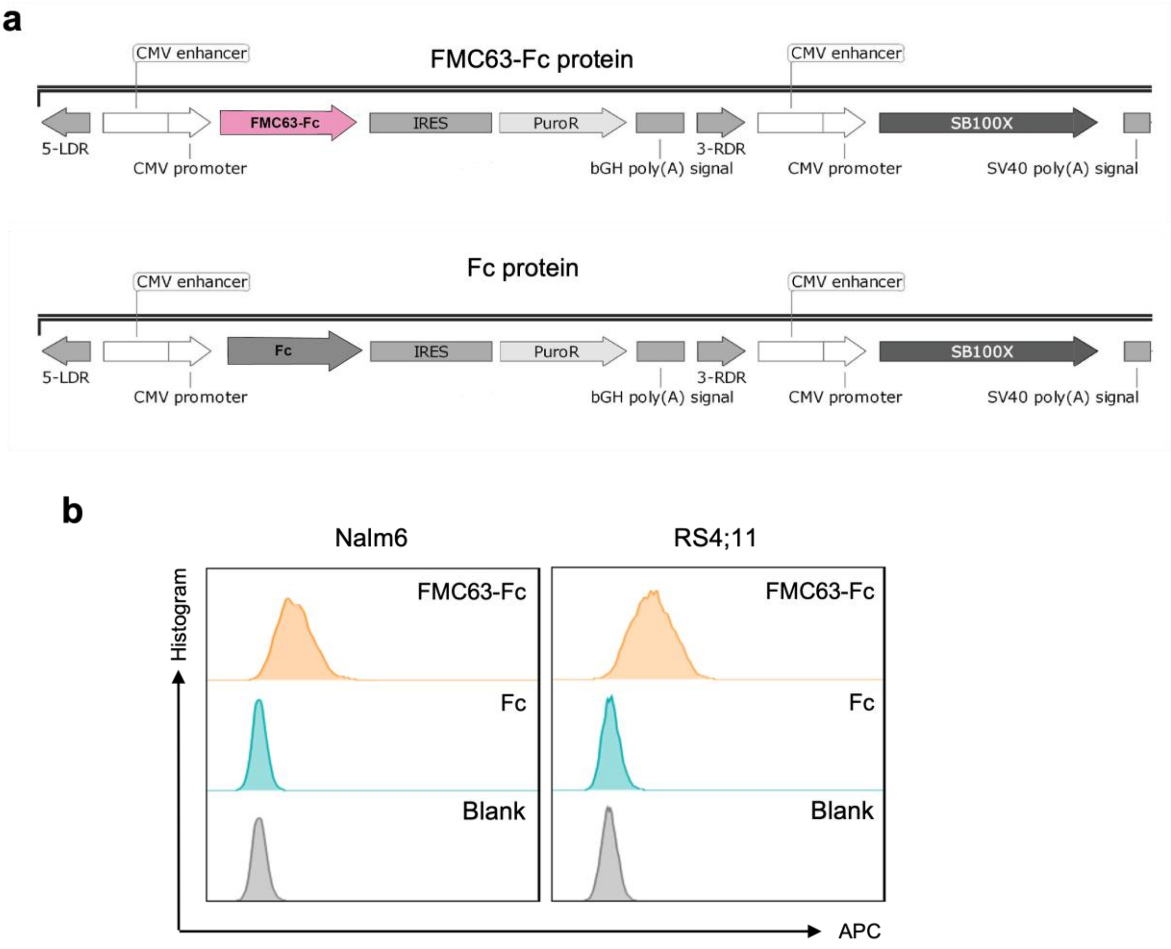

Figure S1. (a) The plasmids for FMC63-Fc and Fc protein expression. (b) Flow cytometry assays to verify binding to BALL cell lines of the expressed proteins.

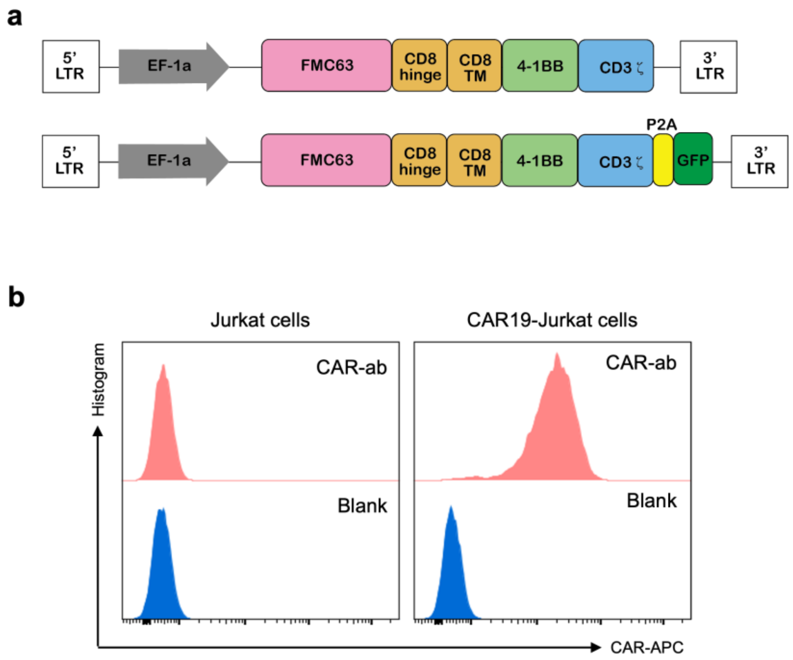

Figure S2. (a) The plasmids for CAR19 and CAR19-GFP. (b) Flow cytometry assays to validate the successful construction of CAR19-Jurkat cells.

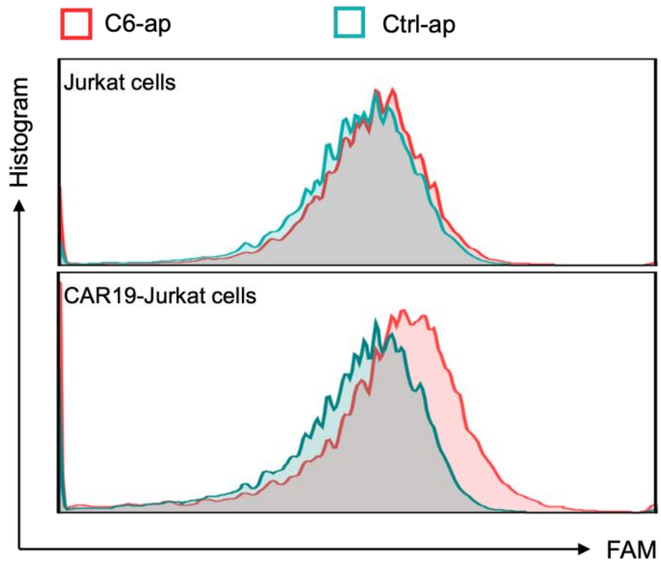

Figure S3. Flow cytometry assay to detect the binding of ssDNA pools (Ctrl-ap and c6-ap) to CAR19-Jurkat cells.

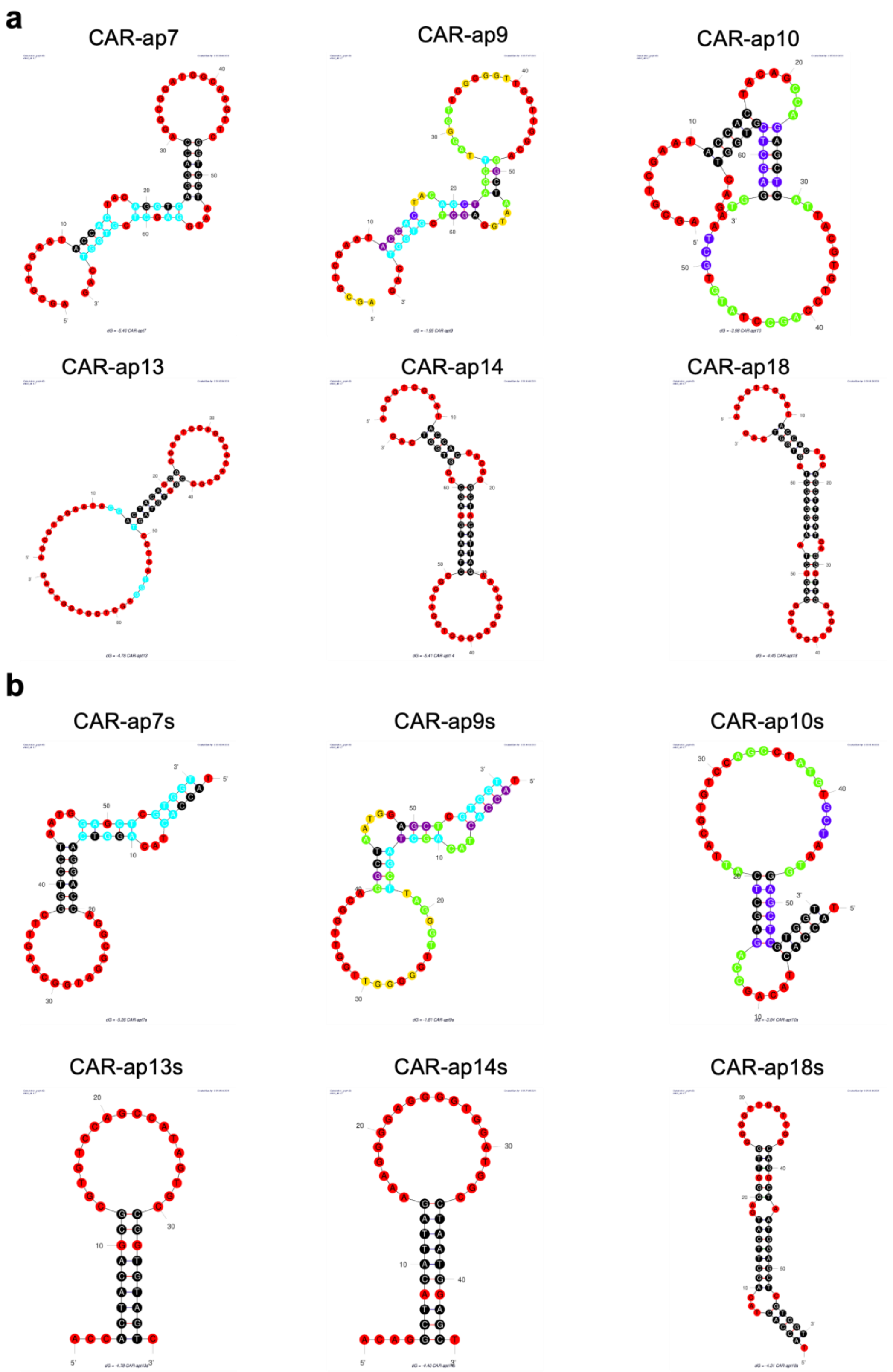
a
CAR-ap7
CAR-ap9
CAR-ap10
CAR-ap13
CAR-ap14
CAR-ap18
b
CAR-ap7s
CAR-ap9s
CAR-ap10s
CAR-ap13s
CAR-ap14s
CAR-ap18s

Figure S4. The predicted secondary structures of full-length CAR-ap (7, 9, 10, 13, 14, and 18) (a) and optimized CAR-ap (7s, 9s, 10s, 13s, 14s, and 18s) (b) aptamers by Mfold.

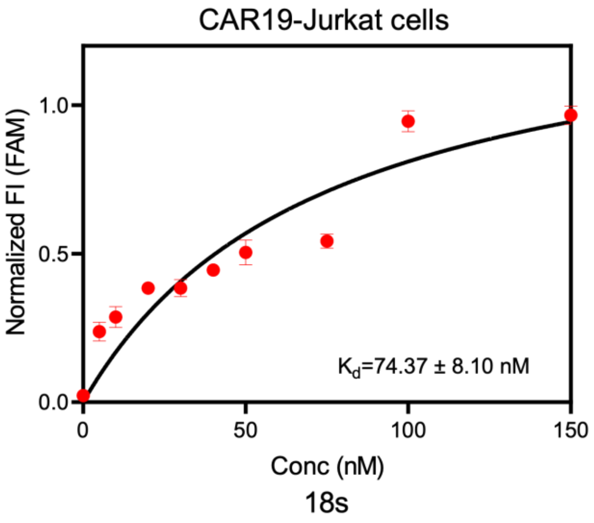

Figure S5. The binding affinity of CAR-ap18s to CAR19-Jurkat cells.

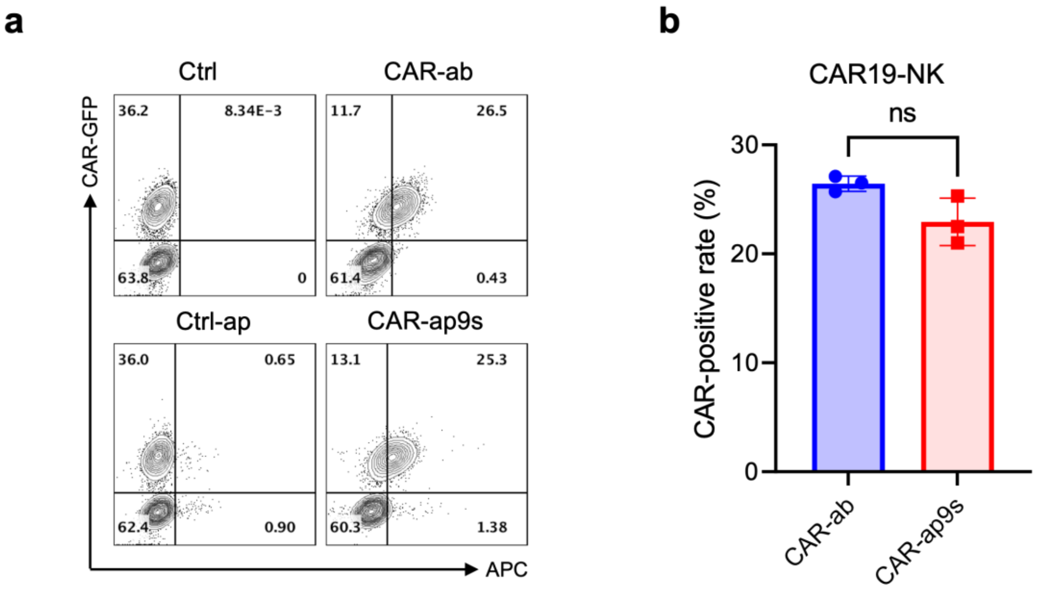

Figure S6. Flow cytometry assay of CAR-ab and CAR-ap9s aptamer binding to CAR19-NK92 cells. Left, flow cytometry plots representing three replicates (a). Right, the percentage of GFP$^+$ NK92 cells that were also positive for CAR-ab or CAR-ap9s aptamer binding (b).

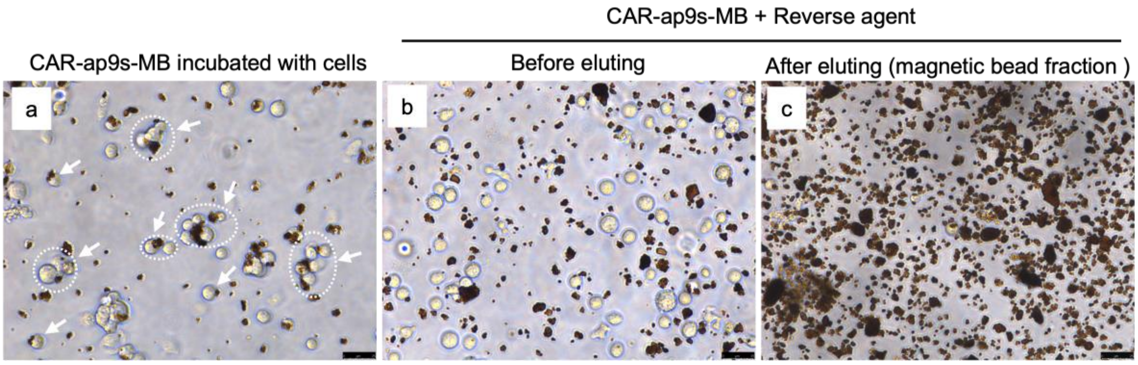

Figure S7. Microscopic observation of CAR19-T cell binding to the magnetic beads during cell sorting. (a) Magnetic beads loaded with aptamers bind to CAR-positive T cells and form cell clusters (white circles and arrows). (b) CAR-positive T cells are released from magnetic beads after adding the reverse agent and the cell clusters disappear. (c) CAR-positive T cells are eluted and completely separated from the magnetic bead fraction.

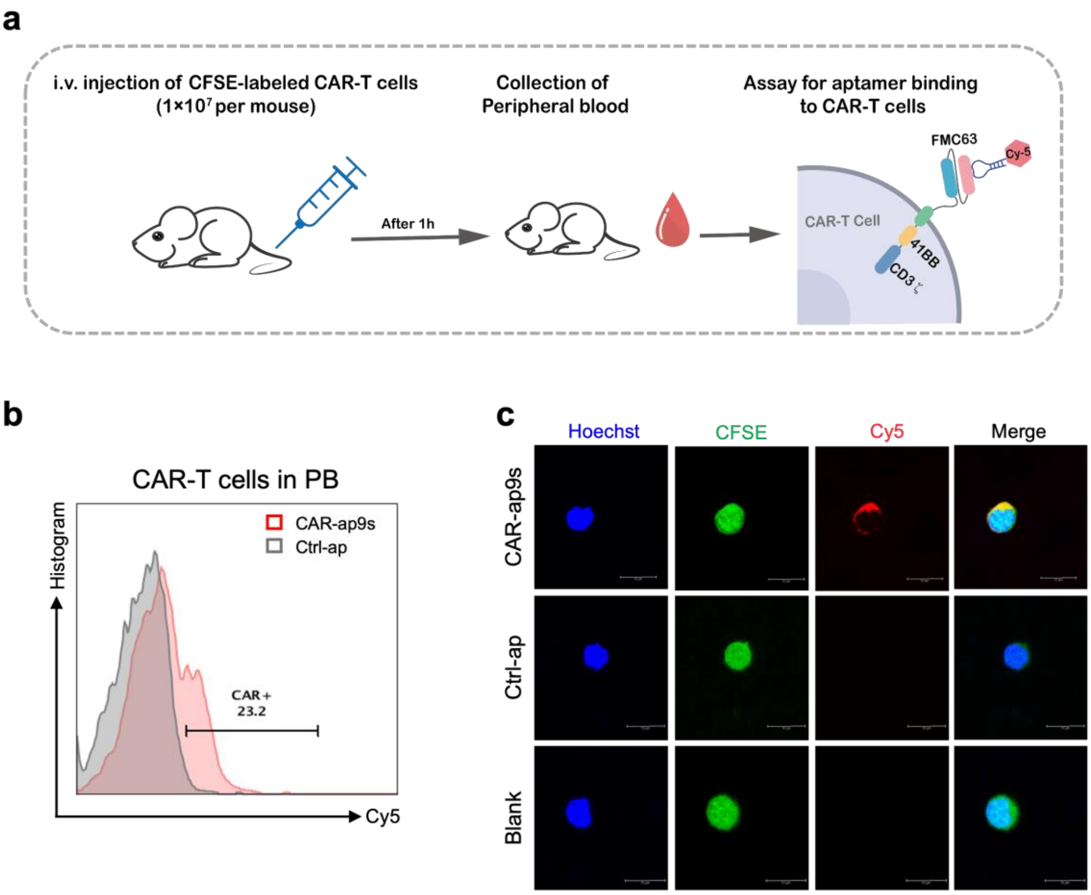

Figure S8. (a) Schematic of CAR-ap9s aptamer binding to CFSE-labeled CAR19-T cells in peripheral blood (PB). (b) Flow cytometry assay to detect CAR-ap9s aptamer and Ctrl aptamer binding to CFSE$^+$ CAR19-T cells in PB. (c) Fluorescence confocal microscopy of PB cells from CFSE-labeled CAR19-T cells injected into a mouse stained with CAR-ap9s-cy5 aptamer or Ctrl-ap-cy5 aptamer.

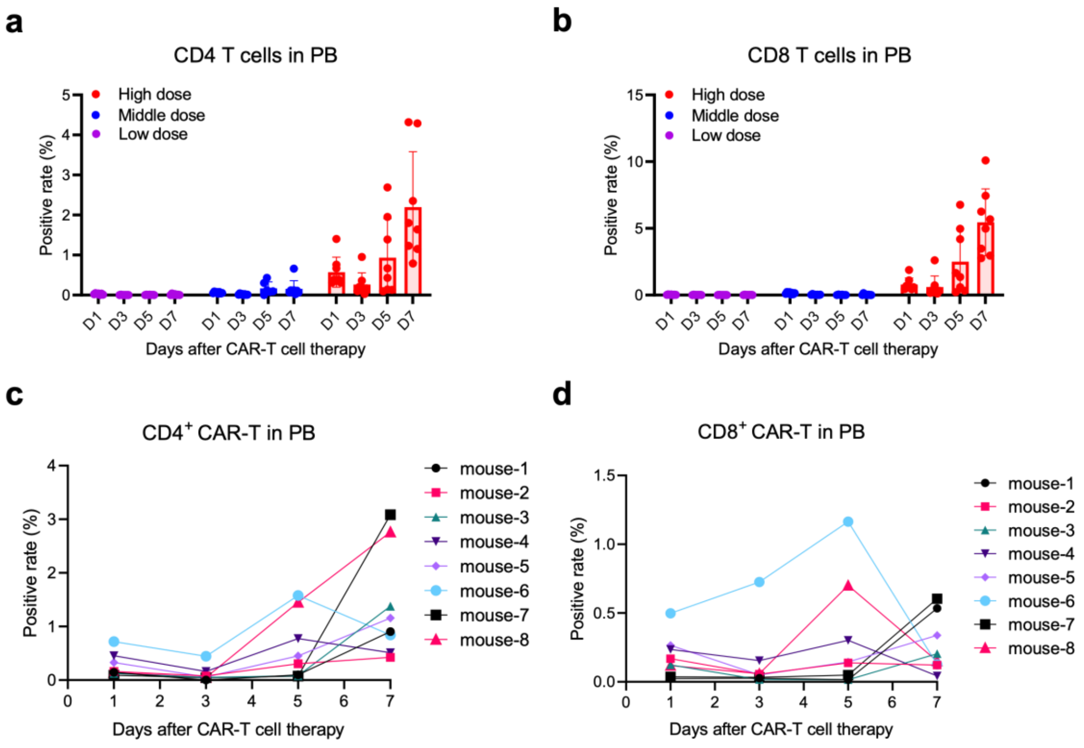

Figure S9. (a-b) Flow cytometry assay of the percentage of $CD4^{+}$ T cells (a) and $CD8^{+}$ T cells (b) in the PB of Nalm6-modeled mice treated with low-doses (purple), middle-doses (blue), and high-doses (red) of CAR19-T cells. (c-d) Flow cytometry detection of the expansion of $CD4^{+}CAR^{+}$ T cells (c) and $CD8^{+}CAR^{+}$ T cells (d) in the PB of each mouse in the high-dose group at different time points.

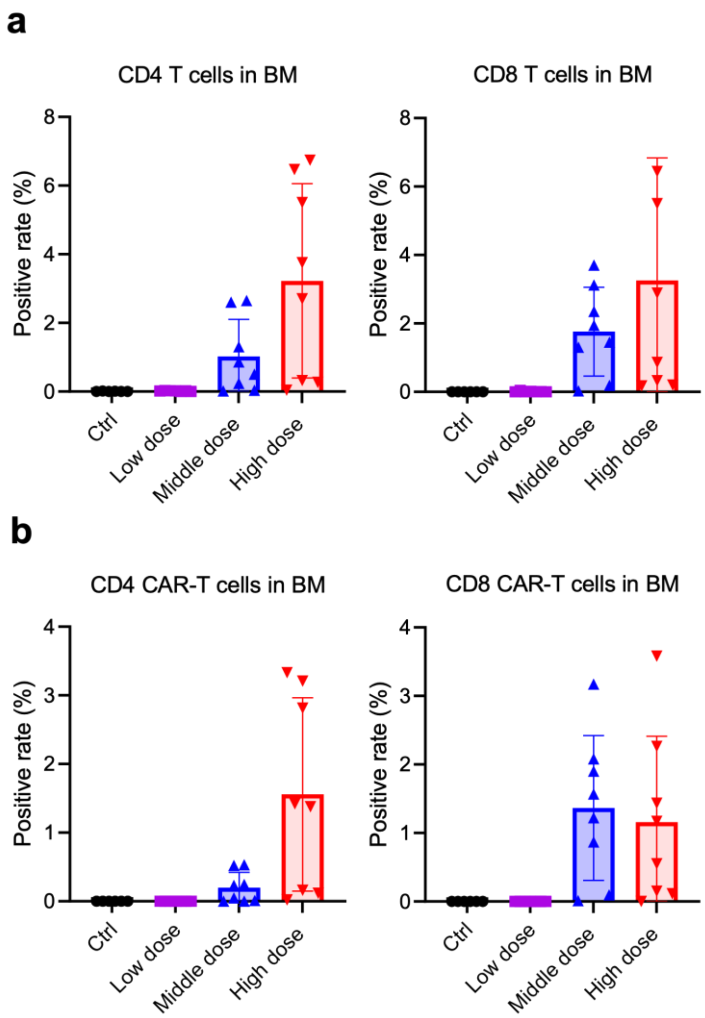

Figure S10. Flow cytometry assay of the percentage of CD4^{+} T cells, CD8^{+} T cells (a), CD4^{+}CAR^{+} T cells, and CD8^{+}CAR^{+} T cells (b) in the bone marrow of Nalm6-modeled mice on the eighth day after treatment with low-dose (purple), middle-dose (blue), and high-dose (red) CAR19-T cells.

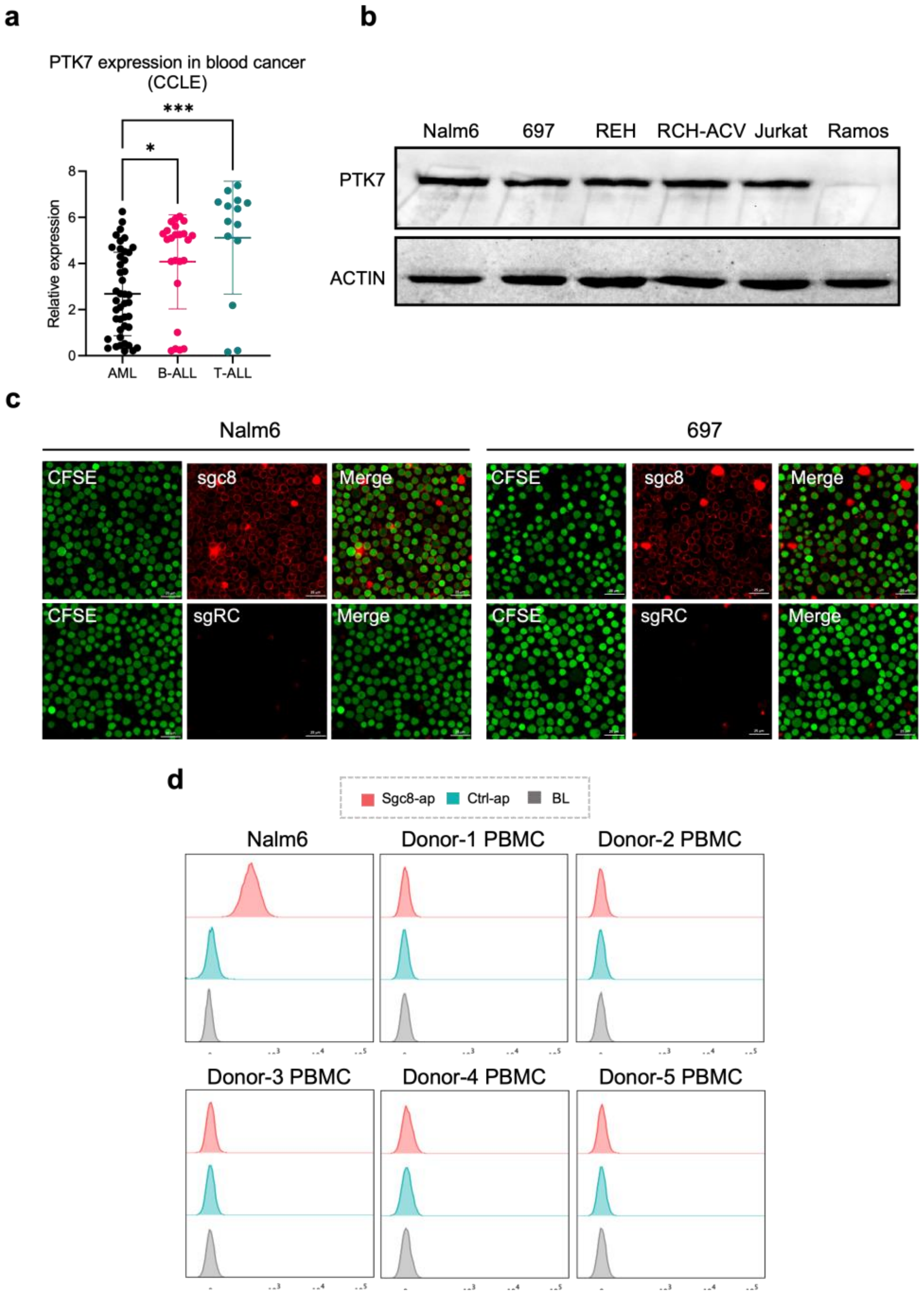

Figure S11. (a) Expression of PTK7 in AML, B-ALL, and T-ALL tumor cell lines and data from the Cancer Cell Line Encyclopedia (CCLE). (b) PTK7 expression in B-ALL cell lines was detected by Western blot. Jurkat cells and Ramos cells were used as positive and negative control, respectively. (c) Fluorescence confocal microscopy of Sgc8-cy5 aptamer binding to Nalm6 cells and 697 cells. (d) Flow cytometry assay of sgc8 aptamer (sgc8-ap) binding to Nalm6 cells and healthy donors' PBMCs.

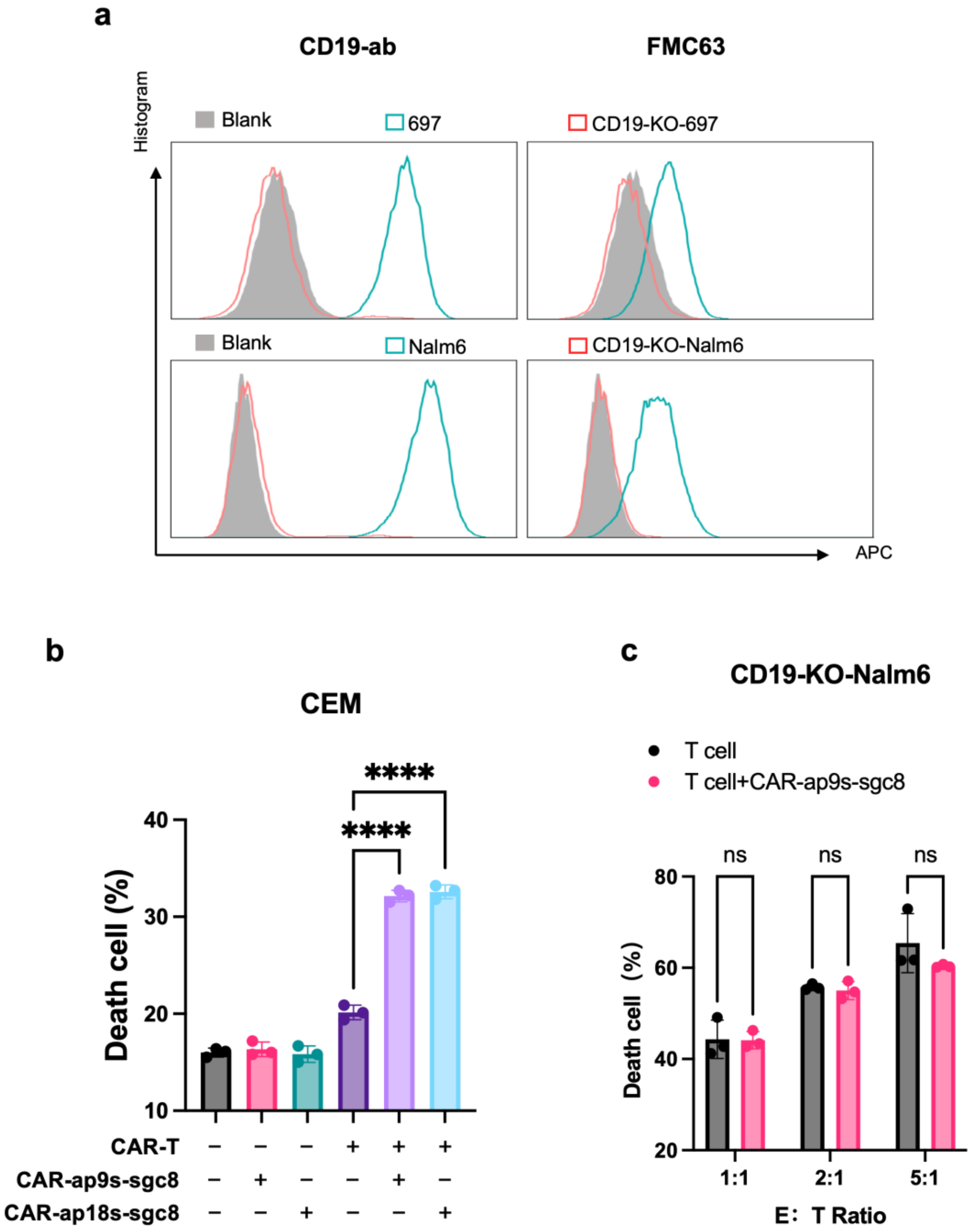

Figure S12. (a) The binding of commercial antibody CD19-ab or self-prepared FMC63 protein to CD19-KO-697 or CD19-KO-Nalm6 cells was determined by flow cytometry. (b) Evaluation of CAR19-T cell killing against CEM cells mediated by CAR-ap9s-sgc8 and CAR-ap18s-sgc8. (c) Detection of basal nonspecific killing of CD19-KO-Nalm6 cells by T cells.

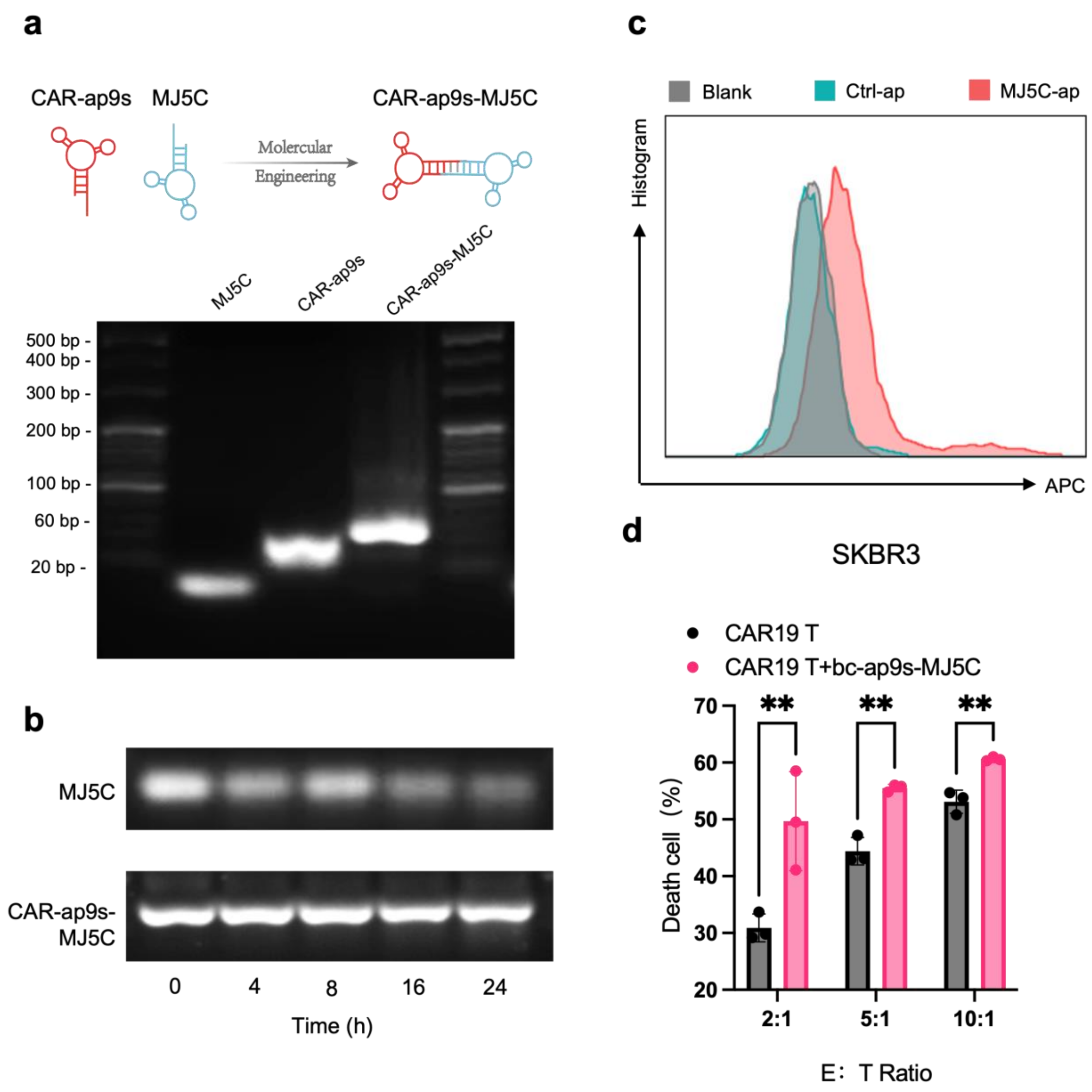

Figures S13. (a) Schematic illustrating the construction of bispecific circular aptamer, CAR-ap-MJ5C. Agarose gel electrophoresis of ssDNA CAR-ap9s, ssDNA MJ5C, and CAR-ap9s-MJ5C. (b) The stability of ssDNA MJ5C and CAR-ap9s-MJ5C in FBS was determined by agarose gel electrophoresis at 37 ℃ for the different incubation times. (c) Flow cytometry assay of the MJ5C aptamer (MJ5C-ap) binding to SKBR3 cells. (d) *In vitro* antitumor cytotoxicity of CAR19-T cells to SKBR3 cells at different E : T ratios in the presence or absence of CAR-ap-MJ5C.

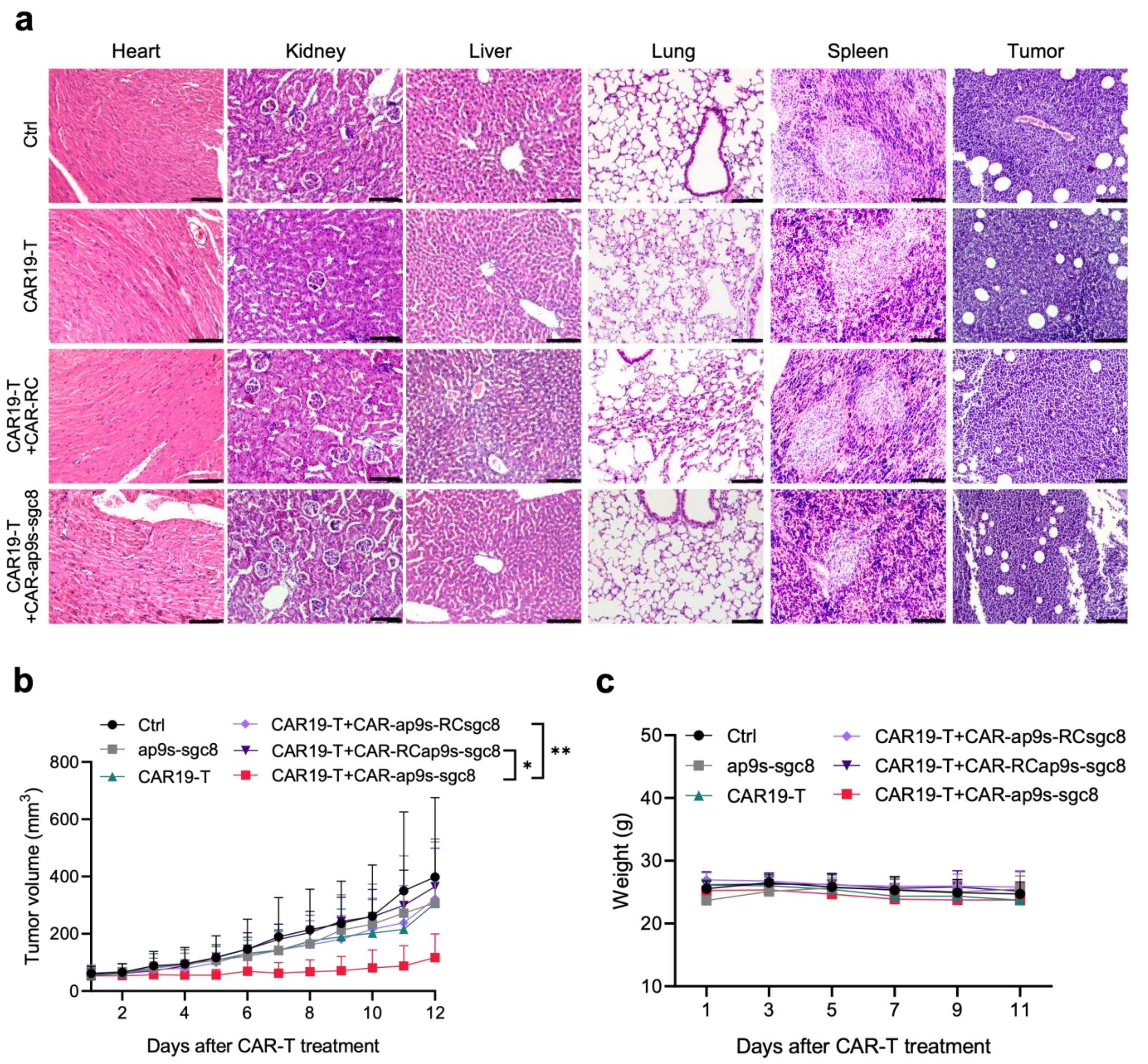

Figure S14. (a) H&E staining of tissue sections from CEM tumor xenografts after treatment. Scale bar: 100 μm. (b) Average tumor growth kinetics of mice under different treatments. Tumor volume (mm^3) was monitored daily using the caliper method. Data are presented as means ± s.d. (n=5). Statistical analyses were performed using two-tailed paired Student's t-tests (*p <0.05, **p<0.01, ***p <0.001, n.s., not significant). NSG mice (n=5 per group) were inoculated in the flank with CD19-KO-Nalm6 tumor cells (1×10^7) on day 14. After two weeks of tumor establishment, CAR19-T cells (1×10^7) were administered intravenously (i.v.) to tumor-bearing NSG mice on day 0. Different groups of mice received different intratumoral (i.t.) treatments on day 1 to day 5 (50μL of PBS for CTRL and CAR19-T groups, bc-RCap9s-sgc8, ap9s-RCsgc8, or bc-ap9s-sgc8). (c) Body weight was monitored and recorded. Data are presented as means ± s.d. (n=5).

Table S1. Sequences in this work

Name	Sequence(5'-3')
**ssDNA pool**	AGCGTCGAATACCACTACAGNNNNNNNNNNNNNNNNNNNNNNNNNNNNNNNNNNNCTAATGGAGCTCGTGGTCAG
**Primer-F**	AGCGTCGAATACCACTACAG
**Primer-R**	Biotin-CTGACCACGAGCTCCATTAG
**CAR-ap7**	AGCGTCGAATACCACTACAGGTCAGGACCAGGCGGATGGCAAGTTCGGTCCTAATGGAGCTCGTGGTCAG
**CAR-ap9**	AGCGTCGAATACCACTACAGCTAGCTTAGGGTTGGGGGTTGGTTGGCAGGCTAATGGAGCTCGTGGTCAG
**CAR-ap10**	AGCGTCGAATACCACTACAGCCAGAGCTCATTACGTGTCCAGCCTATGTGCTAATGGAGCTCGTGGTCAG
**CAR-ap13**	AGCGTCGAATACCACTACAGCGCGTGTCCAGCCATAGTGCCGGTGTAGTCCTAATGGAGCTCGTGGTCAG
**CAR-ap14**	AGCGTCGAATACCACTACAGGCTACATTAGAAAGGGGAGGGGTGGATGGCCTAATGGAGCTCGTGGTCAG
**CAR-ap18**	AGCGTCGAATACCACTACAGCTTCATGAGGGTTGGGGGTTGGTTGGCAGGCTAATGGAGCTCGTGGTCAG
**CAR-ap7s**	TACCACTACAGGTCAGGACCAGGCGGATGGCAAGTTCGGTCCTAATGGAGCTCGTGGT
**CAR-ap9s**	TACCACTACAGCTAGCTTAGGGTTGGGGGTTGGTTGGCAGGCTAATGGAGCTCGTGGT
**CAR-ap10s**	TACCACTACAGCCAGAGCTCATTACGTGTCCAGCCTATGTGCTAATGGAGCTCGTGGT
**CAR-ap13s**	ACCACTACAGCGCGTGTCCAGCCATAGTGCCGGTGTAGTC
**CAR-ap14s**	ACAGGCTACATTAGAAAGGGGAGGGGTGGATGGCCTAATGGAGCT
**CAR-ap18s**	TACCACTACAGCTTCATGAGGGTTGGGGGTTGGTTGGCAGGCTAATGGAGCTCGTGGT

**Sgc8**	GTCTAACTGCTGCGCCGCCGGGAAAATACTGTACGGTTAGAC
**Ctrl-ap（sgRC）**	GTCTAACCGTACAGTATTTTCCCGGCGGCGCAGCAGTTAGAC
**CAR-ap9s-FAM/Cy5**	FAM/Cy5-TACCACTACAGCTAGCTTAGGGTTGGGGGTTGGTTGGCAGGCTAATGGAGCTCGTGGT
**CAR-ap18s-FAM/Cy5**	FAM/Cy5-TACCACTACAGCTTCATGAGGGTTGGGGGTTGGTTGGCAGGCTAATGGAGCTCGTGGT
**Sgc8-FAM/Cy5**	FAM/Cy5-GTCTAACTGCTGCGCCGCCGGGAAAATACTGTACGGTTAGAC
**Ctrl-ap-FAM/Cy5 （sgRC-FAM/Cy5）**	FAM/Cy5-GTCTAACCGTACAGTATTTTCCCGGCGGCGCAGCAGTTAGAC
**sgc8-13L-2-5P-INBIO**	Phosphate-TGACTGAT/iBiodT/TACG GTCTAACTGCTGCGCCGCCGGGAAAATACTGTACGGTTAGAC
**CAR-ap9s-13L-1-5P**	Phosphate-CGTAAATCAGTCA ACCACTACAGCTAGCTTAGGGTTGGGGGTTGGTTGGCAGGCTAATGGAGCTCGTGGT
**MJ5C-13L-1-5P**	Phosphate-CGTAAATCAGTCA ACAGGTTCTGGGGGGTGGGTGGGGAACCTGT

**sgc8-13L-1-5P**	Phosphate-CGTAAATCAGTCA GTCTAACTGCTGCGCCGCCGGGAAAATACTGTACGGTTAGAC
**CAR-ap9S-13L-2-5P**	Phosphate-TGACTGATTTACG ACCACTACAGCTAGCTTAGGGTTGGGGGTTGGTTGGCAGGCTAATGGAGCTCGTGGT
**RC9S-13L-2-5P**	Phosphate-TGACTGATTTACG ACCACGAGCTCCATTAGCCTGCCAACCAACCCCCAACCCTAAGCTAGCTGTAGTGGT
**RCsgc8-13L-1-5P**	Phosphate-CGTAAATCAGTCA GTCTAACCGTACAGTATTTTCCCGGCGGCGCAGCAGTTAGAC

Table S2. Sequences of aptamers and reversal agent (RA) used in the cell sorting experiments

Name	Sequence(5'-3')
**CAR-ap9s-biotin**	Biotin-TACCACTACAGCTAGCTTAGGGTTGGGGGTTGGTTGGCAGGCTAATGGAGCTCGTGGT
**CAR-ap18s-biotin**	Biotin-TACCACTACAGCTTCATGAGGGTTGGGGGTTGGTTGGCAGGCTAATGGAGCTCGTGGT
**RA-CAR-ap9s**	ACCACGAGCTCCATTAGCCTGCCAACCAACCCCCAACCCTAAGCTAGCTGTAGTGGTA

**RA-CAR-ap18s**	ACCACGAGCTCCATTAGCCTGCCAACCAACCCCCAACCCTCATGAAGCTGTAGTGGTA

Table S3. Sequences of CRISPR/cas9 sgRNA for CD19 knockout

Name	Sequence
**sgRNA-CD19-Oligo 1**	CACCGTTCCTCGGGCCTGACTTCCA
**sgRNA-CD19-Oligo 2**	CTGGAAGTCAGGCCCGAGGAACAAA
